# Systematic Assessment of Safety Threshold for Donor Age in Cadaveric Liver Transplantation

**DOI:** 10.3389/fmed.2021.596552

**Published:** 2021-03-04

**Authors:** Wenchao Wang, Zhengtao Liu, Junjie Qian, Jun Xu, Shuping Que, Li Zhuang, Lei Geng, Lin Zhou, Shusen Zheng

**Affiliations:** ^1^Division of Hepatobiliary and Pancreatic Surgery, Department of Surgery, First Affiliated Hospital, School of Medicine, Zhejiang University, Hangzhou, China; ^2^National Health Commission Key Laboratory of Combined Multi-organ Transplantation, Key Laboratory of the Diagnosis and Treatment of Organ Transplantation, Chinese Academy of Medical Sciences, First Affiliated Hospital, School of Medicine, Zhejiang University, Hangzhou, China; ^3^Key Laboratory of Organ Transplantation, First Affiliated Hospital, School of Medicine, Zhejiang University, Hangzhou, China; ^4^Science for Life Laboratory, Kungliga Tekniska Högskolan - Royal Institute of Technology, Stockholm, Sweden; ^5^DingXiang Clinics, Hangzhou, China; ^6^Shulan (Hangzhou) Hospital Affiliated to Zhejiang Shuren University Shulan International Medical College, Hangzhou, China

**Keywords:** liver transplantation, donor age, mortality, outcomes, dose-response analysis

## Abstract

**Background:** Donor age affects allograft quality and the prognosis of recipients after liver transplantation (LT). Clinicians have assessed the quality of grafts from older donors based on their appearance and texture, with no reliable quantitative evidence. Our study aimed to assess the quantitative impact of donor age on post-transplant outcomes and its safety threshold for LT, based on the published literature.

**Methods:** Relevant studies were retrieved from the Embase, PubMed, and ISI Web of Science databases. Pooled dichotomous relative risks (RRs) were calculated using metan. Continuous RRs were calculated using a two-stage random-effects model.

**Results:** Eleven studies including 30,691 LT cases were included for further analysis. For categorical comparison, the RR of death within the first post-transplant year was significantly higher among patients who received grafts from older donors. Similarly, the RR of graft failure (GF) was increased within the 3 years after transplantation. For continuous comparison, advanced donor age affected transplant outcomes in a linear manner (*P* > 0.05). A 10-year increment in donor age was associated with RRs 1.10, 1.12, 1.15, 1.10, and 1.08 for 90-day, 180-day, 1-year, 3-year, and 5-year patient mortality and 1.08, 1.06, 1.10, 1.11, and 1.12, for 90-day, 180-day, 1-year, 2-year, and 3-year GF, respectively (all *P* < 0.05). A spline model showed that transplants using grafts from donors <43 years old were not associated with age-related risks (*P* > 0.05). The risk of GF was increased in subgroups with fewer LT cases, longer cold ischemic time, fewer male donors, and recipients with viral hepatitis (*P* < 0.05).

**Conclusion:** Donor age might affect post-LT outcomes in a dose-dependent manner. The safety threshold for donor age in terms of GF should be lowered to 43 years as an early warning for the guarantee of satisfactory outcomes. Clinicians should weigh the benefits against the risks carefully for patients receiving grafts from older donors. Further studies are warranted to investigate the mechanisms responsible for the relationship between donor age and graft quality.

## Introduction

Liver transplantation (LT) is currently considered as one of the most important curative treatments for end-stage liver disease. Marginally suitable grafts are widely utilized by clinicians to overcome organ shortages (1), and more grafts from older donors are used in line with the aging of the global population. Adam et al. reported that donors older than 60 years increased from 1% in 1989 to 29% in 2009 (2), and data from the United Network for Organ Sharing (UNOS) showed that grafts from older donors (>50 years) increased from 2.4% in 1989 to 29% in 1999 and 33% in 2013 (3). However, older donors might yield inferior post-transplant outcomes (4, 5). The aging of the population and improvements in citizen donation systems suggest that more attention should be paid to the effect of age on donor livers *per se*, and its interactions with other problems (e.g., fatty liver, hepatitis C virus (HCV) infection), on the quality of LT.

Allografts from older donors might lead to a poor prognosis for the recipients after LT (6). In addition, livers from older donors might also have an impact on the incidence of ischemic-type biliary complications and hepatic artery thrombosis (HAT) (7, 8). Grafts from older donors have less capacity for regeneration (9) and higher incidences of ischemia-reperfusion injury (IRI) and HCV recurrence (10–12). Surgeons therefore usually routinely discard livers from donors older than 60 years (13). However, some studies have revealed similar post-transplant prognoses using grafts from older donors (14, 15). Increased use of grafts from older donors might help to solve the organ shortage, and several studies have accordingly evaluated the impacts of older donors on recipient prognosis after LT (12, 16–27); however, the conclusions of these studies have differed due to inconsistencies in recipient age, cold ischemia time (CIT), warm ischemia time (WIT), and the original disease of the recipient.

Despite being a crucial continuous covariate in the evaluation of allograft quality, the dose-response relationship between donor age and post-transplant prognosis has not been systematically evaluated. The safety cutoff for donor age has not been determined, and the decision to use or refuse grafts from older donors has mainly been decided based on the clinician's experience, with a lack of systematic evaluation (27). A meta-analysis of previously published data might thus help to determine the age-related dose-dependent impact of using liver grafts from older donors on post-transplant outcomes (28). We therefore performed a systematic evaluation focusing on the role of donor age on the quality of LT to elucidate the following: (1) time-dependent trends and quantitative risk assessment of donor age as a continuous covariate on post-transplant outcomes; (2) the threshold donor age for safe LT; and (3) covariates affecting the association between donor age and post-transplant outcomes.

## Methods

### Search Strategy

The study was performed strictly according to the criteria of Preferred Reporting Items for Systematic Reviews and Meta-Analyses (PRISMA) (29). The Supplementary PRISMA Checklist presents the details of the reporting items. A literature search of PubMed, Embase, and ISI Web of Science (updated until 20 July 2020) was conducted using the terminologies presented in Supplementary Table 1. The language was limited to English. The search details for each database are described in Supplementary Table 1.

### Inclusion Criteria

We searched for articles assessing the impact of donor age on post-LT prognosis. Included articles were required to meet the following criteria: (1) LT cases categorized into three or more groups by donor age; (2) patient mortality and graft failure (GF) reported or could be evaluated by calculation; and (3) follow-up duration > 90-days.

### Data Extraction

Two authors (JQ and WW) extracted the information from all the included studies according to a unified standardized reporting form. Potential inter-author discrepancies were checked and resolved by a third experienced author (LZ). The following information was collected: (1) general information (author, origin country, publication date, and follow-up days); (2) reasons for LT and causes of death after LT; (3) donor/recipient factors [sex, age, model of end-stage liver disease (MELD) score, body mass index (BMI)]; (4) surgery (operation data and surgical approaches); and (5) outcomes (post-transplant laboratory examination, length of hospitalization/intensive care unit (ICU) stay, patient death, and GF).

Data presented in graphs were extracted using GetData Graph Digitizer software (v 2.26; downloaded from http://getdata-graph-digitizer.com/index.php). Hazard ratios (HRs) and variance were retrieved from the provided data or were calculated based on the data provided. Accurate HRs were acquired from Kaplan–Meier curves using Engauge Digitizer (version 4.1) (30, 31). For studies that only provided a range of data, the median value was defined as the mid-point between the upper and lower limits. For open-ended data, the median value was 20% higher than the lower limit or 20% lower than the upper limit.

### Quality Assessment

The quality of the methods in each study was examined by WW and ZL based on the Newcastle-Ottawa Scale (NOS) for non-randomized cohort studies (NOS checklist) (32). We conducted a quality assessment for each included study according to the following three items: (1) patient selection; (2) study comparability; and (3) definition of outcomes of interest. A study with at least six stars was considered as high quality, according to the NOS system (33).

### Data Synthesis

We assessed the risk of donor age on patient mortality, organ failure, and post-transplant complications using HRs and relative risks (RRs). Patients were divided into three or four groups, according to donor age, as indicated in the studies. Donors in the top tertile or highest two quartiles were defined as older, while donors in the lowest tertile/quartile were defined as younger. For categorical comparison, we evaluated the RR based on donor age (older/middle vs. younger). Pooled standardized mean differences were used to evaluate quantitative differences across groups classified by donor age (older/middle vs. younger), and the relationship between donor age and the risk of post-transplant outcomes was shown using a dose-response model. We evaluated the standardized mean differences (SMDs) of body mass index (BMI) based on donor age (older vs. younger) using the metan.

### Subgroup Analysis

We also conducted subgroup analyses to assess the effects of potential confounders on the impacts of donor age on patient/graft survival, classified according to sex distribution (recipient/donor), recipient age/etiology, recipient MELD score, sample size, CIT, WIT, type of study (multicenter vs. single center), year of LT, and origin country.

### Meta-Regression

The effects of intermediate confounders (recipient/donor sex distribution, recipient age/MELD score/etiology, sample size, number of medical centers, CIT/WIT, year of LT, origin country) on the association between donor age and post-transplant outcomes were examined by meta-regression (34).

### Sensitivity Analysis

Sensitivity analysis of categorical/continuous risks of donor age on patient mortality, GF, primary graft non-function (PNF), and re-transplantation was performed to evaluate the influence of each study on the overall pooled results.

### Publication Bias Analysis

Egger's test was used to evaluate the effects of potential publication bias on the categorical/continuous impact of donor age on patient mortality, GF, PNF, and re-transplantation (35).

### Statistical Analysis

Descriptive data with a normal distribution were presented as mean ± standard deviation and compared by one-way ANOVA. Non-normally distributed data were presented as median (inter-quartile range) and compared by Mann–Whitney *U*-tests. Distributions between different groups were compared by χ^2^ test. All analyses were performed using SPSS software (v22.0, SPSS Inc., Chicago, IL, USA).

RRs and corresponding 95% confidence intervals (CIs) were calculated using an online calculator (https://www.medcalc.org/calc/oddsratio.php) if the data were unavailable. We examined statistical heterogeneity using χ^2^ Q and *I*^2^ tests, with *I*^2^ values of 25, 50, and 75% defined as low, moderate, and high heterogeneity, respectively (36). Calculations were carried out using the metan command (37) in Stata software (release 22; Stata-Corp, College Station, TX, USA), and a *P* < 0.05 was considered statistically significant.

The dose-response relationship between donor age and post-transplant outcomes was assessed using a two-stage random-effects dose-response model developed by Orsini et al. (28). Continuous RRs were also evaluated using a restricted cubic spline model. Generalized least squares regression was first used to construct the restricted cubic spline model considering the log RR and relevant variance in each study (38). A multivariate random-effects model was then used to combine each RR (39), and risk tendencies were shown by plotting the pooled RRs. The null hypothesis on regression coefficients was used to examine the evidence for non-linearity in the pooled cubic splines (equal to zero). *P* < 0.05 was considered as significant for a non-linear relationship. For non-linear cubic splines, the safety threshold was defined as the donor age corresponding to the lower 95% CI of RR at 1.

## Result

### Study Extraction

**Figure 1:**
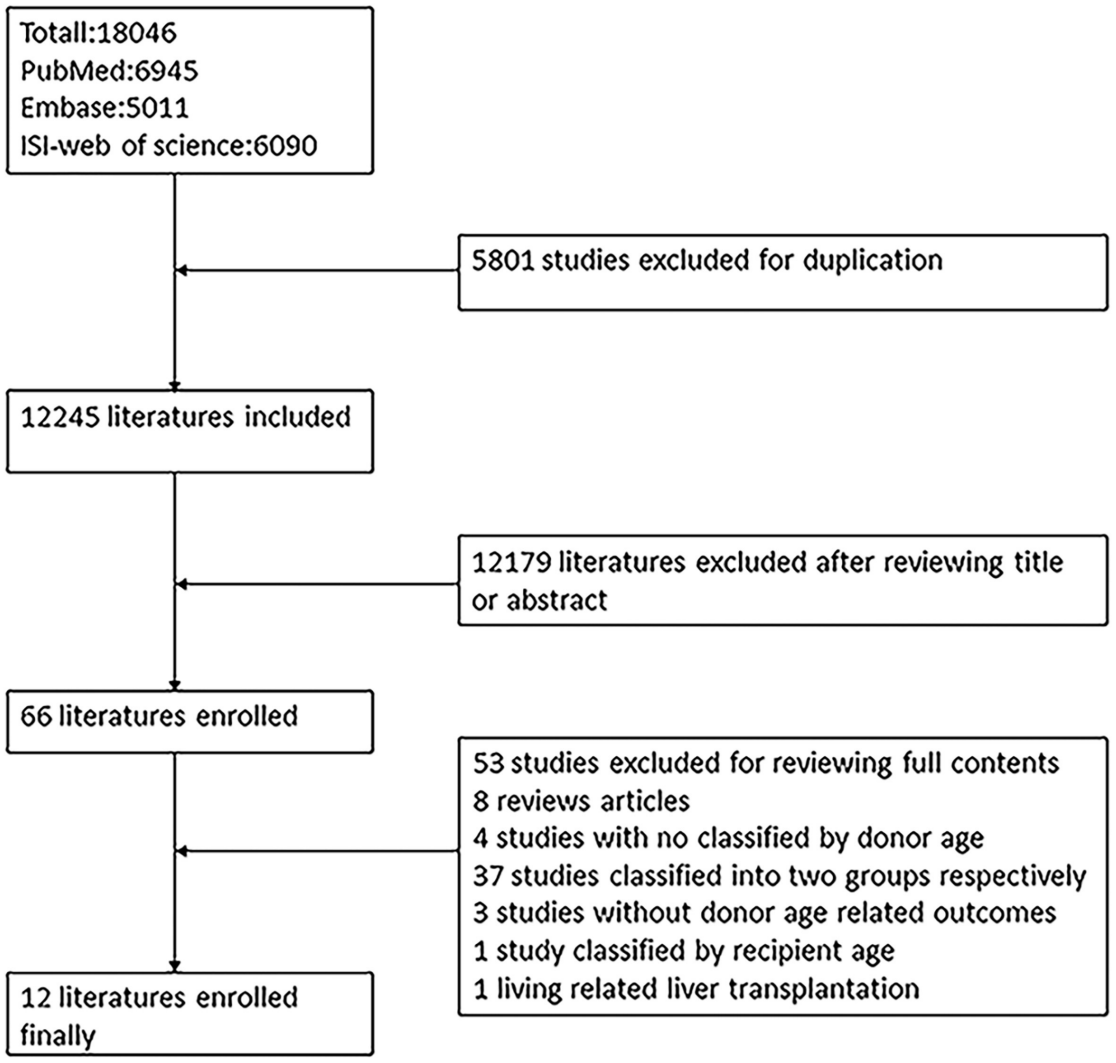
Flow diagram on selection of eligible studies.

The process of literature extraction is shown in Figure 1. Graphical abstract on research scheme is provided in Supplementary Figure 1. A total of 18,046 articles were retrieved from PubMed, Embase, and ISI Web of Science for screening. Eleven articles including clinical studies with information on the effects of donor age on the outcomes after LT were included in the final study, after excluding studies that did not meet the required criteria.

### Quality Assessment

The quality of the included literature was assessed by the NOS (Supplementary Table 2). Eleven studies scored more than six points and exhibited high quality. One study (11) scored four points because of low representativeness of the selected population, absence of MELD score/CIT, and an inadequate follow-up duration, which was excluded.

### Characteristics of Included Studies

The characteristics of the included studies are shown in Supplementary Table 6. Eleven studies including 30,691 patients were included in the meta-analysis. All included LT cases used livers donated after cardiac death or brain death. Five articles (12, 18, 19, 23, 25) included cases from the USA and six (16, 17, 20–22, 24) included cases from Europe. All enrolled LTs were performed between 1986 and 2012. The mean age of the recipients ranged from 48.5 to 60 years. The mean follow-up duration was 6–62 months. Seven studies (16, 17, 20–23, 25) described the sex distribution of the recipients, and the proportion of males was <50% in only one study (23). Eight studies (12, 16, 17, 19, 20, 22, 23, 25) reported the primary liver disease necessitating transplantation, including HCV infection, alcoholic liver cirrhosis, primary bile cirrhosis, and hepatocellular carcinoma. Data in five studies (12, 18, 21–23) were derived from multiple centers and data in six studies (16, 17, 19, 20, 24, 25) were from single center. Details of donor age were provided for each subgroup in each study.

Post-transplant outcomes for each study are shown in Supplementary Table 3. Donor-age-related recipient survival was reported in seven studies (16–18, 21–24), and donor-age-related graft survival was reported in nine studies (12, 16, 17, 19–23, 25). The mean donor age ranged from 30 to 65 years in all enrolled studies. Four studies provided data on PNF (16, 17, 21, 22), two studies reported on the length of hospitalization and ICU stay (16, 21), three provided information on re-transplantation (16, 17, 21), and one study each reported data on HAT (21) and ischemic type biliary lesions (ITBL) (16).

### Categorical Comparison Between Donor Age and Post-transplant Outcomes

**Figure 2:**
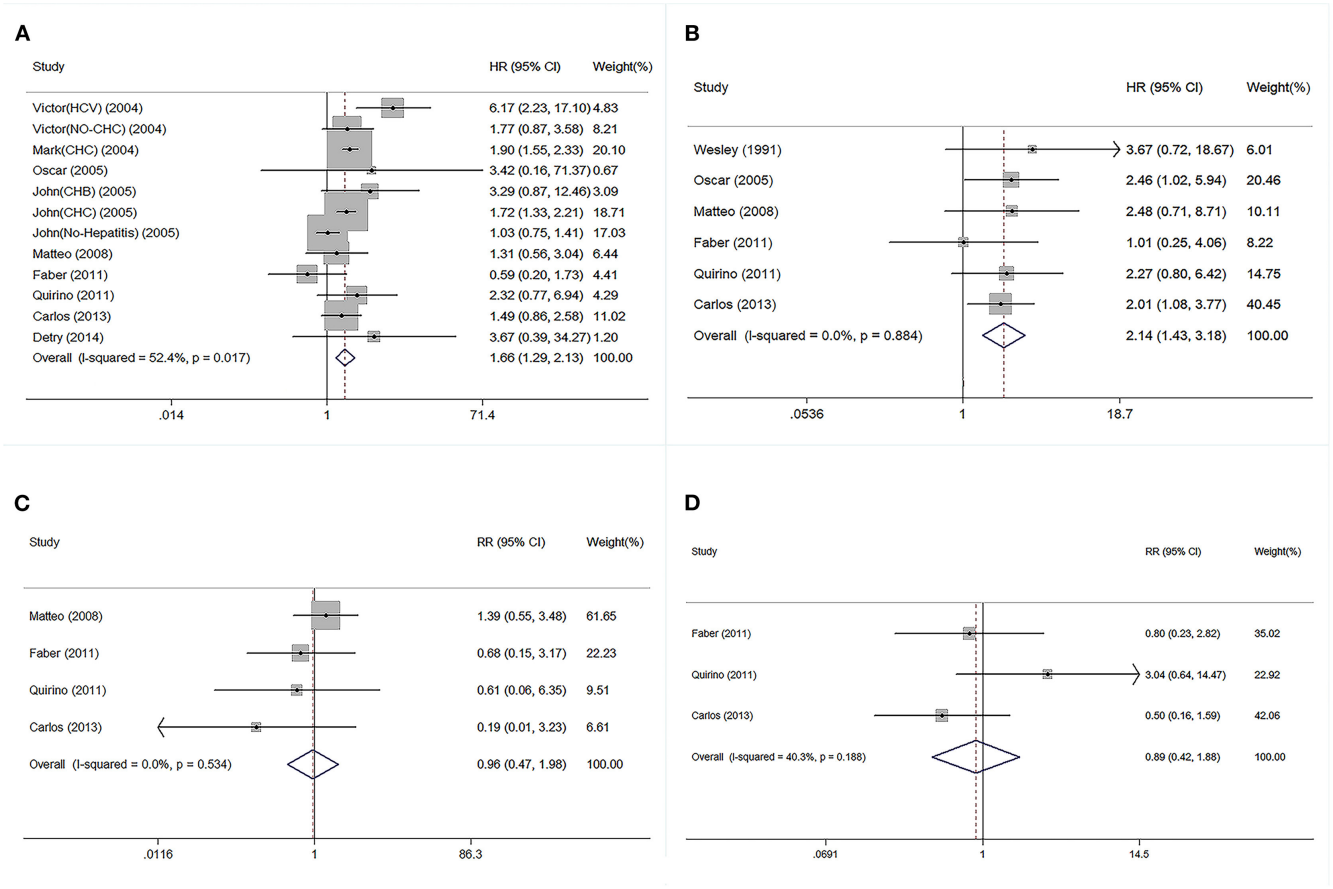
Forest plot on association between donor age and post-transplant outcomes. **(A)** Pooled RR of graft failure by comparison between elder and younger donor groups. **(B)** Pooled RR of patient death by comparison between elder and younger donor groups. **(C)** Pooled RR of PNF by comparison between elder and younger donor group. **(D)** Pooled RR of re-transplantation by comparison between elder and younger donor groups. PNF, primary non-function; RR, relative risk.

The HR for GF increased from 0.59 to 6.17 in the subgroup of patients receiving older compared with younger donor livers, and the pooled HR for GF was 1.66 (Figure 2A). The HR for patient mortality was increased from 1.01 to 3.67 in the subgroup receiving older donor livers (Figure 2B), and the pooled HR for patient mortality was 2.14 (*P* < 0.05, Figure 2B). The pooled RR of 1-year patient mortality was significantly higher in patients receiving older grafts (RR = 1.76, 95% CI: 1.28–2.43), while the impact of allograft age on graft loss lasted longer, and the pooled HR for 3-year GF was significantly higher (RR = 1.72, 95% CI: 1.51–1.96) (Table 1).

**Table 1:** Comparison on post-operational outcomes categorized by donor age.

**Item**	**Comparison**	**Number of studies**	**Number of patients**	**Pooled RR**	***I^**2**^(%)***	***P* for heterogeneity^**a**^**	***P* for egger's test^**b**^**	**Incidence (%)^**c**^**
**Patient mortality**								
90-days	Older vs. younger	6	910	1.46 (0.94–2.27)	0	0.48	0.08	9.01
	Middle vs. younger	6	1,171	1.40 (1.01–1.95)	4.20	0.39	0.92	10.42
180-days	Older vs. younger	7	910	1.38 (1.15–1.65)	8.00	0.37	0.37	13.30
	Middle vs. younger	7	1,171	1.14 (0.99–1.33)	49.30	0.07	0.30	15.29
1-year	Older vs. younger	6	910	1.76 (1.28–2.43)	62.50	0.02	0.18	16.85
	Middle vs. younger	6	1,171	1.66 (1.30–2.12)	44.20	0.11	0.77	19.47
2-years	Older vs. younger	4	600	1.16 (0.97–1.39)	0	0.45	0.74	23.33
	Middle vs. younger	4	786	1.09 (0.94–1.28)	4.50	0.37	0.22	24.04
3-years	Older vs. younger	4	600	1.14 (0.97–1.36)	0	0.57	0.22	25.67
	Middle vs. younger	4	785	1.18 (1.04–1.33)	57.30	0.05	0.32	28.50
5-years	Older vs. younger	4	600	1.16 (0.99–1.37)	68.00	0.03	0.55	31.33
	Middle vs. younger	4	786	1.17 (1.04–1.32)	29.90	0.23	0.46	32.44
**Graft failure**								
90-dayss	Older vs. younger	7	7,576	1.11 (0.97–1.28)	46.80	0.04	0.19	13.25
	Middle vs. younger	7	8,657	1.14 (1.01–1.29)	57.90	0.01	0.66	12.80
180-day	Older vs. younger	9	10,738	1.36 (1.24–1.50)	49.10	0.03	0.78	17.10
	Middle vs. younger	8	8,657	1.13 (1.01–1.25)	68.40	<0.01	0.59	15.60
1-year	Older vs. younger	8	7,601	1.41 (1.27–1.55)	66.50	<0.01	0.6	20.62
	Middle vs. younger	8	8,538	1.30 (1.19–1.43)	79.60	<0.01	0.82	19.40
2-years	Older vs. younger	7	7,410	1.51 (1.39–1.65)	62.90	<0.01	0.69	25.74
	Middle vs. younger	8	8,567	1.30 (1.20–1.41)	69.10	<0.01	0.39	23.60
3-years	Older vs. younger	6	3,212	1.72 (1.51–1.96)	70.70	<0.01	0.62	26.56
	Middle vs. younger	6	4,115	1.22 (1.07–1.38)	68.80	<0.01	0.55	22.41
5-years	Older vs. younger	3	552	1.27 (0.95–1.69)	68.80	0.04	0.87	32.43
	Middle vs. younger	3	715	1.24 (0.98–1.57)	67.10	0.05	0.90	32.90
PNF	Older vs. younger	4	788	0.96 (0.47–1.98)	0	0.53	0.05	5.08
	Middle vs. younger	4	880	0.92 (0.47–1.81)	0	0.51	0.48	5.34
Re-transplantation	Older vs. younger	3	504	0.89 (0.42–1.88)	40.30	0.19	0.01	9.72
	Middle vs. younger	3	575	1.02 (0.54–1.93)	53.00	0.12	0.74	9.91

*PNF, primary non-function; RR, relative risk*.

a *P value represented the heterogeneity of pooled results*.

b *P value represented the publication bias of pooled results*.

c *Incidence represented the rate of patient death, graft failure, PNF and re-transplantation for enrolled studies*.

*I^2^Variation in RR attributable to heterogeneity*.

**Figure 3:**
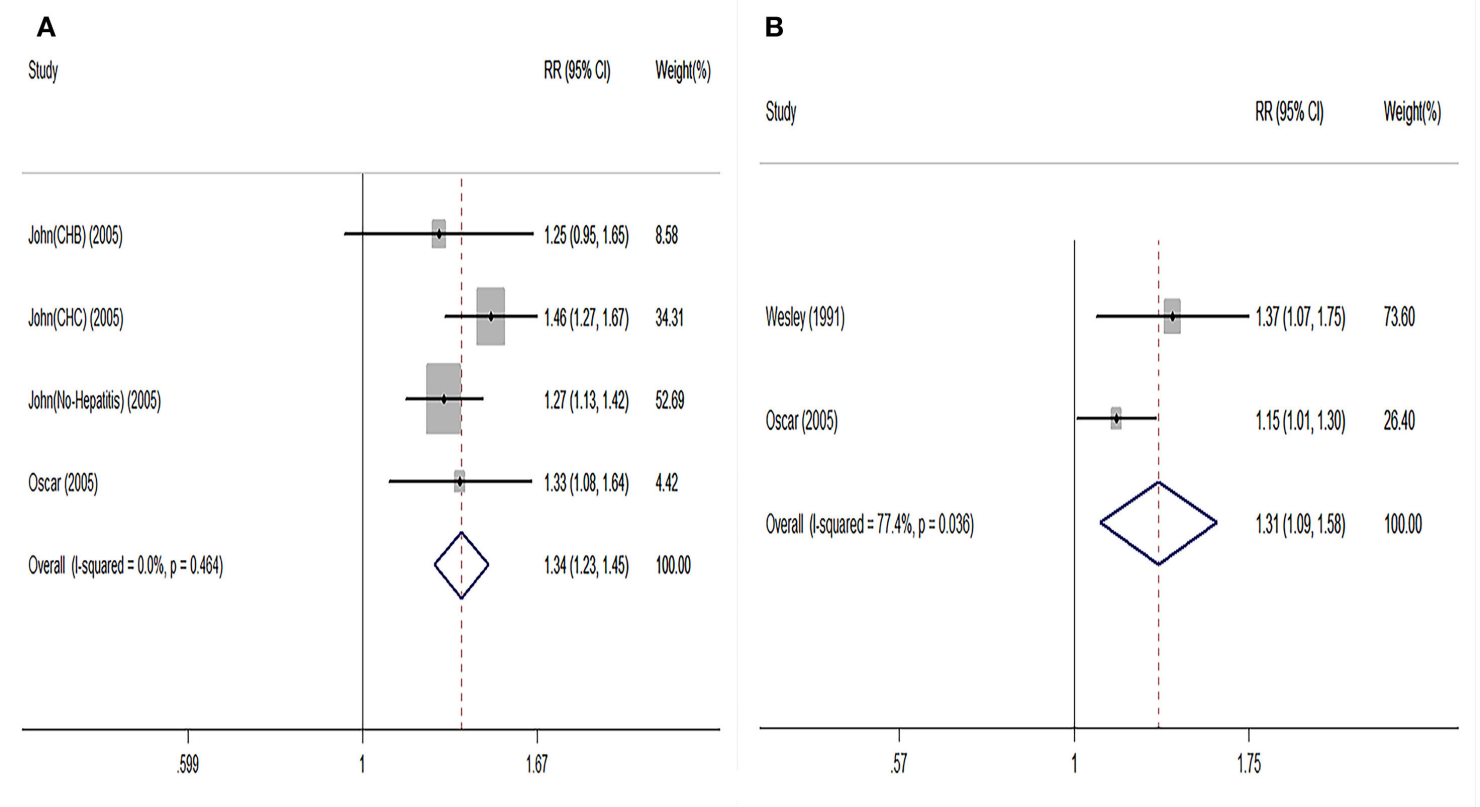
Pooled results on comparison in LT cases categorized by grafts from adults and adolescents. **(A)** Pooled RR on graft failure between adult and adolescent groups. **(B)** Pooled RR on patient death between adult and adolescent groups. Adults were defined as the donors aged elder than 18 years; adolescents were defined as the donors aged younger than 18 years. RR, relative risk; LT, liver transplantation.

Regarding short-term post-transplant complications, the use of older donor livers did not increase the incidence of PNF (pooled RR = 0.96) (Figure 2C). Furthermore, there was no significant association between donor age and re-transplant incidence (RR = 0.89) (Figure 2D). Compared with LTs using adolescent donors (<18 years), the pooled RR for GF was 1.34 (95% CI: 1.23–1.45) (Figure 3A) and the pooled RR for patient death was 1.31 (95% CI: 1.09–1.58) (Figure 3B).

Regarding the time-dependent prognosis in relation to donor age, the risks of mortality in patients receiving a graft from an older donor were significantly increased after 180 days (RR = 1.38, 95% CI: 1.15–1.65) and 1 year (RR = 1.76, 95% CI: 1.28–2.43). However, the effect of using an older liver was reduced after prolonged follow-up, and there was no significantly increased risk of death associated with an older donor in long-term survivors (≥2 years, all *P* > 0.05). The trends in donor-age-related RRs of patient death across different time points are shown in Table 2. RRs of mortality decreased with time (RR = 1.76, 1.16, 1.14, 1.16 at 1, 2, 3, and 5 years, *P* < 0.05). In terms of donor-age-related risk of GF, the RR increased in the first 3 years after LT (RR = 1.11, 1.36, 1.41, 1.51, 1.72 at 90 and 180 days, and 1, 2, and 3 years, *P* < 0.05) (Tables 1, 2), but declined at 5 years (RR = 1.27, 95% CI: 0.95–1.69, *P* > 0.05). Donor age had no significant effect on the RRs of PNF and re-transplantation.

**Table 2:** Comparison on donor age related post-operational outcomes in different time points.

**Item**	**Number of studies**	**Number of patients**	**Pooled RR**	***P* for heterogeneity^**a**^**	**Incidence (%)^**b**^**
**Patient mortality**					
180-day/90-day	7/6	910/910	1.38 (1.15–1.65)/1.46 (0.94–2.27)	0.82	8.14/9.01
1-year/90-day	6/6	910/910	1.76 (1.28–2.43)/1.46 (0.94–2.27)	0.50	16.85/9.01
2-year/90-day	4/ 6	600/910	1.16 (0.97–1.39)/1.46 (0.94–2.27)	0.35	23.33/9.01
3-year/90-day	4/6	600/910	1.14 (0.97–1.36)/1.46 (0.94–2.27)	0.31	25.67/9.01
5-year/90-day	4/6	600/910	1.16 (0.99–1.37)/1.46 (0.94–2.27)	0.34	31.33/9.01
1-year/180-day	6/7	910/910	1.76 (1.28–2.43)/1.38 (1.15–1.65)	0.19	16.85/8.14
2-year/180-day	4/7	600/910	1.16 (0.97–1.39)/1.38 (1.15–1.65)	0.18	23.33/8.14
3-year/180-day	4/7	600/910	1.14 (0.97–1.36)/1.38 (1.15–1.65)	0.13	25.67/8.14
5-year/180-day	4/7	600/910	1.16 (0.99–1.37)/1.38 (1.15–1.65)	0.16	31.33/8.14
2-year/1-year	4/6	600/910	1.16 (0.97–1.39)/1.76 (1.28–2.43)	0.01	23.33/16.85
3-year/1-year	4/6	600/910	1.14 (0.97–1.36)/1.76 (1.28–2.43)	0.02	25.67/16.85
5-year/1-year	4/6	600/910	1.16 (0.99–1.37)/1.76 (1.28–2.43)	0.02	31.33/16.85
3-year/2-year	4/4	600/600	1.14 (0.97–1.36)/1.16 (0.97–1.39)	0.89	25.67/23.33
5-year/2-year	4/4	600/600	1.16 (0.99–1.37)/1.16 (0.97–1.39)	0.98	31.33/23.33
5-year/3-year	4/4	600/600	1.16 (0.99–1.37)/1.14 (0.97–1.36)	0.90	31.33/25.67
**Graft failure**					
180-day/90-day	9/7	10,738/7,576	1.36 (1.24–1.50)/1.11 (0.97–1.28)	0.02	17.10/13.25
1-year/90-day	8/7	7,601/7,576	1.41 (1.27–1.55)/1.11 (0.97–1.28)	<0.01	20.62/13.25
2-year/90-day	7/7	7,410/7,576	1.51 (1.39–1.65)/1.11 (0.97–1.28)	<0.01	25.74/13.25
3-year/90-day	6/7	3,212/7,576	1.72 (1.51–1.96)/1.11 (0.97–1.28)	<0.01	26.56/13.25
5-year/90-day	3/7	552/7,576	1.27 (0.95–1.69)/1.11 (0.97–1.28)	0.43	32.43/13.25
1-year/180-day	8/9	7,601/10,738	1.41 (1.27–1.55)/1.36 (1.24–1.50)	0.52	20.62/17.10
2-year/180-day	9/9	7,410/10,738	1.51 (1.39–1.65)/1.36 (1.24–1.50)	0.11	25.74/17.10
3-year/180-day	6/9	3,212/10,738	1.72 (1.51–1.96)/1.36 (1.24–1.50)	<0.01	26.56/17.10
5-year/180-day	3/9	552/10,738	1.27 (0.95–1.69)/1.36 (1.24–1.50)	0.65	32.43/17.10
2-year/1-year	9/8	7,410/7,601	1.51 (1.39–1.65)/1.41 (1.27–1.55)	0.27	25.74/20.62
3-year/1-year	6/8	3,212/7,601	1.72 (1.51–1.96)/1.41 (1.27–1.55)	0.02	26.56/20.62
5-year/1-year	3/8	552/7,601	1.27 (0.95–1.69)/1.41 (1.27–1.55)	0.50	32.43/20.62
3-year/2-year	6/9	3,212/7,410	1.72 (1.51–1.96)/1.51 (1.39–1.65)	0.12	26.56/25.74
5-year/2-year	3/9	552/7,410	1.27 (0.95–1.69)/1.51 (1.39–1.65)	0.25	32.43/25.74
5-year/3-year	3/6	552/3212	1.27 (0.95–1.69)/1.72 (1.51–1.96)	0.06	32.43/26.56

*RR, relative risk*.

a *P represented the heterogeneity between subgroups*.

b *Incidence represented the rate of patient death and graft failure for included studies*.

Regarding post-transplant complications, the risk of ITBL was increased in patients receiving older allografts (RR = 7.16, 95% CI: 1.50–34.27, *P* < 0.05) (Supplementary Figure 2). However, there was no difference in the incidence of HAT in relation to donor age (*P* > 0.05) (Supplementary Figure 2), and no significant increase in the risk of length of hospitalization and ICU stay (*P* > 0.05) (Supplementary Figure 3). The pooled BMI was higher in the older donors compared to youngers (SMD = 0.39, 95% CI: 0.20–0.58, *P* < 0.05; Supplementary Figure 4). The information of donors with diabetes was only reported in one study and it showed elder donors had a higher prevalence of diabetes compared to younger donors (*P* < 0.05, Supplementary Figure 5) (22).

### Continuous Impacts of Donor Age on Post-transplant Outcomes

**Figure 4:**
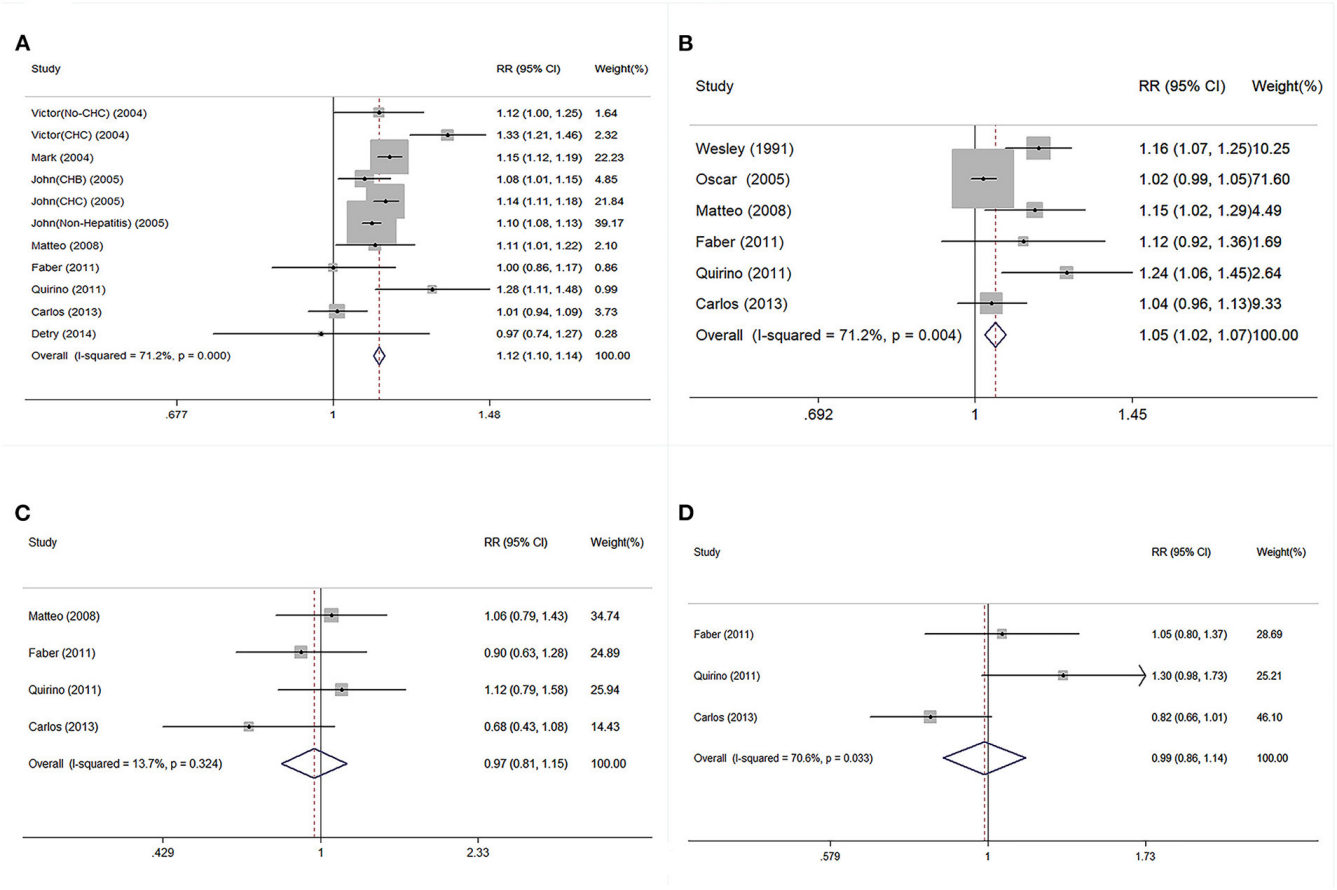
Continuous risk of post-transplant outcomes was evaluated followed per 10-year increment on donor age in linear model. **(A)** Pooled RR of GF followed per 10-year increment on donor age. **(B)** Pooled RR of patient death followed per 10-year increment on donor age. **(C)** Pooled RR of PNF followed per 10-year increment on donor age. **(D)** Pooled RR of re-transplantation followed per 10-year increment on donor age. RR, relative risk; GF, graft failure; PNF, primary non-function.

The pooled continuous risks of donor age on patient outcomes are presented in Figure 4. The RRs of a 10-year increment in donor age on graft loss, patient death, PNF, and re-transplantation were 1.12 (95% CI: 1.10–1.14), 1.05 (95% CI: 1.02–1.07), 0.97 (95% CI: 0.81–1.15), and 0.99 (95% CI: 0.86–1.14), respectively (Figure 4). Dose-response analysis using a generalized least-squares regression model revealed that donor age affected patient outcomes in linear manner (*P* for non-linearity > 0.05) (Table 3). The continuous risk of donor age on post-transplant outcomes is shown in Table 3. The continuous risk of donor age increment on patient mortality was increased up to the 1st year (RR = 1.10, 1.12, and 1.15 at 90 days, 180 days, and 1 year, respectively), but declined to borderline significance at 3 and 5 years (RR = 1.10 and 1.08 at 3 and 5 years, respectively) (Table 3). The continuous RR of donor age increment on GF was increased up to 3 years after LT, but the increased risk was no longer significant by 5 years (Table 3). There was no significant association between donor age and the incidence of PNF or re-transplantation (both *P* > 0.05) (Table 3).

**Table 3:** Continuous risk of donor age on post-transplant outcomes in linear model.

**Item**	**Number of studies**	**Number of participants**	***P* for non-linearity**	**RR (95CI)^**a**^**	***P* for heterogeneity^**b**^**	***I*^**2**^(%)**
Patient mortality				GLS		
90-day	6	3,421	0.54	1.10 (1.04–1.17)	0.19	20.50
180-day	6	3,421	0.16	1.12 (1.07–1.16)	0.43	2.10
1-year	6	3,421	0.72	1.15 (1.08–1.22)	0.02	40.80
2-year	6	1,195	0.67	1.06 (0.99–1.13)	0.86	0
3-year	4	1,195	0.21	1.10 (1.00–1.21)	0.11	32.00
5-year	4	1,161	0.41	1.08 (1.00–1.16)	0.24	19.60
Graft failure				GLS		
90-day	8	20,753	0.24	1.08 (1.04–1.11)	<0.01	46.80
180-day	8	20,753	0.24	1.06 (1.05–1.15)	<0.01	60.90
1-year	8	20,753	0.93	1.10 (1.05–1.13)	<0.01	65.10
2-year	7	20,481	0.57	1.11 (1.07–1.16)	<0.01	67.20
3-year	6	14,077	0.50	1.12 (1.08–1.16)	<0.01	72.00
5-year	3	1,092	0.94	1.12 (0.98–1.27)	0.10	36.20
PNF	4	1,364	0.95	0.96 (0.80–1.16)	0.83	0
Re-transplantation	3	811	0.97	1.03 (0.79–1.34)	0.19	30.40

*GLS, generalized least square; PNF, primary non-function; RR, relative risk*.

a *RR represented the risk of adverse events followed per 10 years increment on donor age*.

b *P value represented the heterogeneity of pooled results*.

### Safety Threshold for Donor Age in Cadaveric LT

**Figure 5:**
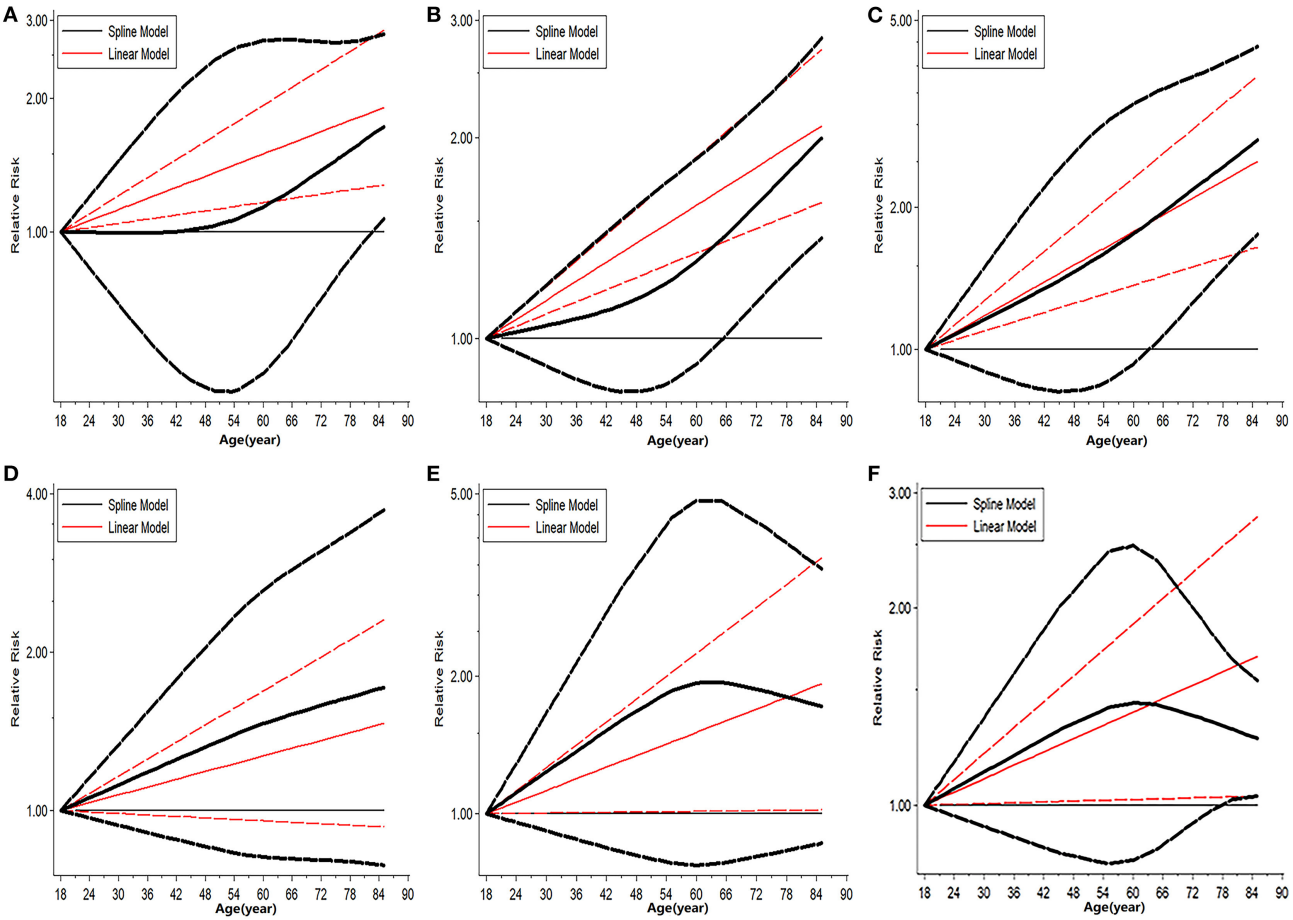
Dose-response analysis on risk of advanced donor age on patient death. **(A)** Dose-response risk of advanced donor age on 90-day patient death. **(B)** Dose-response risk of advanced donor age on 180-day patient death. **(C)** Dose-response risk of advanced donor age on 1-year patient death. **(D)** Dose-response risk of advanced donor age on 2-year patient death. **(E)** Dose response risk of advanced donor age on 3-year patient death. **(F)** Dose response risk of advanced donor age on 5-year patient death.

**Figure 6:**
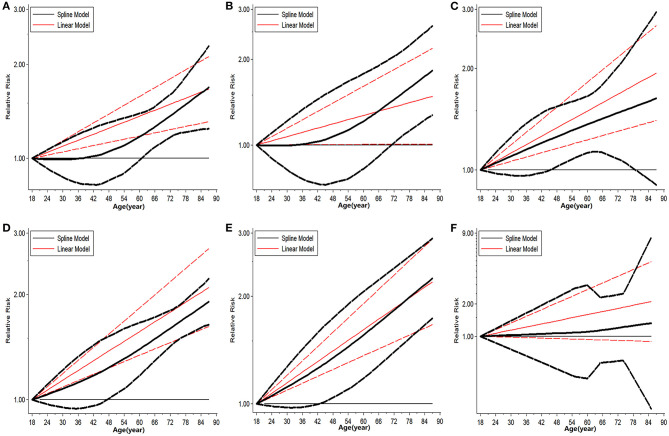
Dose response analysis on risk of advanced donor age on GF. **(A)** Dose-response risk of advanced donor age on 90-day GF. **(B)** Dose-response risk of advanced donor age on 180-day GF. **(C)** Dose-response risk of advanced donor age on 1-year GF. **(D)** Dose-response risk of advanced donor age on 2-year GF. **(E)** Dose-response risk of advanced donor age on 3-year GF. **(F)** Dose-response risk of advanced donor age on 5-year GF. GF, graft failure.

**Figure 7:**
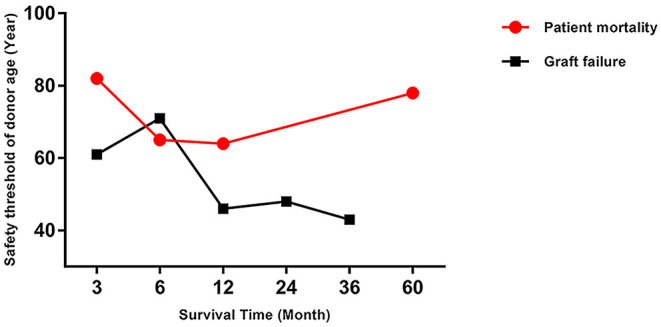
Safety threshold of donor age on patient death and GF at different time-points. GF, graft failure.

The trend in risk of patient mortality related to donor age at different time-points is shown in Figures 5, 6 and the dose-response relationship is shown in Table 4. The risk thresholds for 90-day, 180-day, 1-year, and 5-year patient mortality in the spline model were 82, 65, 64, and 78 years, respectively (Table 4, Figure 5). There was no intersection on the spline model fitted for donor age-related 2-year and 3-year patient mortality (Table 4, Figure 5). Concerning allograft loss, the safety thresholds for 90-day, 180-day, 1-year, 2-year, and 3-year graft loss in the spline model were 61, 71, 46, 48, and 43 years, respectively (Table 4, Figure 6), and there was no intersection for donor- age-related 5-year GF in the dose-response spline model (Table 4, Figure 6). The safety thresholds for donor age in relation to patient death/GF at different time-points are presented in Figure 7 and detailed in Table 4. Donor age had no significant impact on PNF or re-transplantation incidence at all time points (Table 4, Supplementary Figure 6).

**Table 4:** Risk of donor age on post-operational outcomes in different time points by non-linear model.

**Item**	**Number of studies**	**Number of participants**	**Pooled RR (95% CI)^**a**^**	**P for heterogeneity^**b**^**	**P for significance^**c**^**	**Age safety threshold (%)^**d**^**
**Patient mortality**						
90-day	6	3,421	RCS	0.19	0.13	82
20 vs. 18 y			1.05 (0.94–1.12)			
30 vs. 18 y			0.98 (0.67–1.58)			
40 vs. 18 y			0.99 (0.51–1.68)			
50 vs. 18 y			1.04 (0.44–2.28)			
60 vs. 18 y			1.15 (0.49–2.53)			
70 vs. 18 y			1.32 (0.65–2.74)			
80 vs. 18 y			1.58 (0.92–2.74)			
180-day	6	3,421	RCS	0.42	<0.01	65
20 vs. 18 y			1.01 (0.99–1.03)			
30 vs. 18 y			1.04 (0.88–1.22)			
40 vs. 18 y			1.09 (0.84–1.38)			
50 vs. 18 y			1.17 (0.85–1.53)			
60 vs. 18 y			1.30 (0.92–1.77)			
70 vs. 18 y			1.53 (1.08–2.13)			
80 vs. 18 y			1.84 (1.30–2.52)			
1-year	6	3,421	RCS	0.02	<0.01	64
20 vs. 18 y			1.02 (0.99–1.04)			
30 vs. 18 y			1.13 (0.88–1.51)			
40 vs. 18 y			1.29 (0.82–2.10)			
50 vs. 18 y			1.51 (0.83–2.74)			
60 vs. 18 y			1.75 (0.94–3.26)			
70 vs. 18 y			2.12 (1.17–3.75)			
80 vs. 18 y			2.51 (1.55–4.19)			
2-year	6	1,195	RCS	0.86	0.02	Na
20 vs. 18 y			1.02 (0.99–1.05)			
30 vs. 18 y			1.12 (0.94–1.32)			
40 vs. 18 y			1.23 (0.89–1.73)			
50 vs. 18 y			1.35 (0.85–2.18)			
60 vs. 18 y			1.46 (0.82–2.67)			
70 vs. 18 y			1.57 (0.81–3.06)			
80 vs. 18 y			1.67 (0.80–3.49)			
3-year	4	1,195	RCS	0.11	<0.01	Na
20 vs. 18 y			1.32 (0.98–1.13)			
30 vs. 18 y			1.25 (0.92–1.70)			
40 vs. 18 y			1.50 (0.85–2.71)			
50 vs. 18 y			1.72 (0.81–3.80)			
60 vs. 18 y			1.89 (0.78–4.51)			
70 vs. 18 y			1.92 (0.79–4.71)			
80 vs. 18 y			1.80 (0.83–3.82)			
5-year	4	1,161	RCS	0.25	<0.01	78
20 vs. 18 y			1.02 (0.98–1.05)			
30 vs. 18 y			1.14 (0.93–1.41)			
40 vs. 18 y			1.25 (0.86–1.81)			
50 vs. 18 y			1.35 (0.83–2.21)			
60 vs. 18 y			1.41 (0.86–2.32)			
70 vs. 18 y			1.41 (0.91–2.15)			
80 vs. 18 y			1.32 (1.02–1.75)			
**Graft mortality**						
90-day	8	20,753	RCS	<0.01	0.61	61
20 vs. 18 y			1.01 (0.98–1.03)			
30 vs. 18 y			0.97 (0.84–1.15)			
40 vs. 18 y			1.01 (0.82–1.24)			
50 vs. 18 y			1.07 (0.86–1.31)			
60 vs. 18 y			1.17 (0.99–1.38)			
70 vs. 18 y			1.33 (1.13–1.60)			
80 vs. 18 y			1.52 (1.23–1.91)			
180-day	8	20,753	RCS	<0.01	0.05	71
20 vs. 18 y			1.01 (0.98–1.02)			
30 vs. 18 y			0.98 (0.81–1.23)			
40 vs. 18 y			1.03 (0.73–1.38)			
50 vs. 18 y			1.11 (0.75–1.62)			
60 vs. 18 y			1.21 (0.83–1.75)			
70 vs. 18 y			1.38 (0.97–2.01)			
80 vs. 18 y			1.63 (1.15–2.37)			
1-year	8	20,753	RCS	<0.01	<0.01	46
20 vs. 18 y			1.01 (0.96–1.12)			
30 vs. 18 y			1.10 (0.94–1.26)			
40 vs. 18 y			1.18 (0.98–1.39)			
50 vs. 18 y			1.27 (1.05–1.51)			
60 vs. 18 y			1.36 (1.10–1.71)			
70 vs. 18 y			1.45 (1.11–1.91)			
80 vs. 18 y			1.55 (0.99–2.43)			
2-year	7	20,481	RCS	<0.01	<0.01	48
20 vs. 18 y			1.02 (0.99–1.04)			
30 vs. 18 y			1.07 (0.73–1.24)			
40 vs. 18 y			1.15 (0.82–1.41)			
50 vs. 18 y			1.26 (1.04–1.53)			
60 vs. 18 y			1.39 (1.18–1.65)			
70 vs. 18 y			1.56 (1.36–1.79)			
80 vs. 18 y			1.75 (1.54–2.00)			
3-year	6	14,077	RCS	<0.01	0 <0.01	43
20 vs. 18 y			1.02 (0.99–1.04)			
30 vs. 18 y			1.11 (0.96–1.29)			
40 vs. 18 y			1.23 (0.98–1.55)			
50 vs. 18 y			1.38 (1.06–1.81)			
60 vs. 18 y			1.56 (1.18–2.08)			
70 vs. 18 y			1.78 (1.35–2.38)			
80 vs. 18 y			2.04 (1.56–2.69)			
5-year	3	1,092	RCS	0.10	<0.01	Na
20 vs. 18 y			1.01 (0.96–1.11)			
30 vs. 18 y			1.02 (0.68–2.25)			
40 vs. 18 y			1.03 (0.55–2.32)			
50 vs. 18 y			1.07 (0.52–2.00)			
60 vs. 18 y			1.12 (0.54–1.89)			
70 vs. 18 y			1.17 (0.52–2.71)			
80 vs. 18 y			1.27 (0.41–5.03)			
PNF	4	1,364	RCS	0.83	0.93	Na
20 vs. 18 y			0.99 (0.92–1.15)			
30 vs. 18 y			0.95 (0.59–1.54)			
40 vs. 18 y			0.91 (0.40–2.00)			
50 vs. 18 y			0.88 (0.31–2.75)			
60 vs. 18 y			0.85 (0.28–2.70)			
70 vs. 18 y			0.84 (0.29–2.22)			
80 vs. 18 y			0.85 (0.30–2.82)			
Re-transplantation	3	811	RCS	0.18	0.76	Na
20 vs. 18 y			0.99 (0.93–1.04)			
30 vs. 18 y			0.98 (0.65–1.49)			
40 vs. 18 y			0.97 (0.46–2.11)			
50 vs. 18 y			0.96 (0.34–2.69)			
60 vs. 18 y			0.95 (0.27–3.13)			
70 vs. 18 y			0.92 (0.25–3.30)			
80 vs. 18 y			0.90 (0.26–2.98)			

*PNF, primary non-function; RCS, Restricted cubic spline*.

a *Pooled RR was the risk of different donor age on patient prognosis*.

b *P-value represented the heterogeneity across included studies*.

c *P-value represented the statistical significance on pooled RRs of enrolled studies*.

d *Threshold was evaluated at the value of donor age responded to 1 at lower 95% CI for spline model*.

### Subgroup Analysis

The risk of donor-age-related patient mortality differed significantly when classified by year of LT (Table 5). The RR was higher in studies including more recent procedures (1.45 vs. 1.13, *P* < 0.05) (Table 5), and the mean donor age was higher in more recent studies (57.7 vs. 46.4 years). The effects of donor age on GF differed significantly between subgroups categorized by the number of study centers, length of CIT, donor sex distribution, and recipient etiology (all *P* < 0.05). The risk of donor-age-related GF was higher in single-center studies (RR = 1.37, *P* < 0.01) (Table 5), in studies with more grafts from female donors (<60 vs. >60% male donors; 1.38 vs. 0.98, *P* < 0.05) (Table 5), and in cases with prolonged CIT (>350 min, *P* < 0.05) (Table 5). Notably, the effect of increased donor age on GF lost its significance in cases with shorter CIT (RR = 0.97, 95% CI: 0.76–1.23, *P* = 0.80) (Table 5), as validated in subgroup analysis. Similarly, studies including recipients with chronic hepatitis B (CHB) or chronic hepatitis C (CHC) had higher risks of donor-age-related GF (RR = 1.52, 95% CI: 1.26–1.84 for CHB; RR = 1.38, 95% CI: 1.31–1.45 for CHC) (Table 5). No single factor had any significant impact on the incidence of PNF, and the effects of aging donors on PNF and re-transplantation were still insignificant in subgroup analysis (*P* > 0.05) (Table 5).

**Table 5:** Subgroup analysis on dose-response risk of donor age on post-transplant outcomes.

	**Number of studies**	**Number of patients**	**RR (95% CI)**	***I*^**2**^ (%)**	***P* for heterogeneity^**a**^**	***P* for heterogeneity^**b**^^***s***^**
**Patient mortality**						
**Sample size**						
< =300	3	575	1.15 (1.06–1.24)	38.90	0.11	
>300	3	2,665	1.26 (1.09–1.45)	41.60	0.08	0.26
**Study center**						
Single-center	3	575	1.15 (1.06–1.24)	38.90	0.11	
Multi-center	3	2,665	1.26 (1.09–1.45)	41.60	0.08	0.26
**Recipient MELD score**						
< =15	2	460	1.67 (1.16–2.39)	41.80	0.13	
>15	2	904	1.28 (1.07–1.53)	32.80	0.19	0.20
**Cold ischemic time (min)**						
< =400	2	539	1.14 (1.06–1.24)	35.90	0.15	
>400	2	825	1.27 (1.21–1.34)	51.90	0.08	0.95
**Warm ischemic time (min)**						
< =50	1	272	1.19 (0.72–1.97)	43.60	0.16	
>50	2	539	1.36 (1.05–1.75)	51.90	0.08	0.65
**Gender of recipient (male%)**						
< =70	2	623	1.15 (0.89–1.48)	16.70	0.31	
>70	2	741	1.51 (1.22–1.86)	41.80	0.11	0.11
**Gender of donor (male%)**						
< =60	2	460	1.67 (1.16–2.39)	41.80	0.13	
>60	2	904	1.28 (1.07–1.53)	32.80	0.19	0.20
**Recipient age (y)**						
< =52	2	904	1.28 (1.07–1.53)	32.80	0.19	
>52	2	460	1.67 (1.16–2.39)	41.80	0.13	0.20
**Year for liver transplantation**						
1989–2003	3	2,227	1.13 (1.05–1.22)	16.10	0.30	
2006–2009	3	1,013	1.45 (1.20–1.77)	38.90	0.10	0.02
**Study region**						
European	5	1,479	1.17 (1.08–1.26)	35.60	0.08	
USA	1	1,761	1.23 (0.97–1.55)	61.90	0.05	0.70
**Graft failure**						
**Sample size**						
< =500	6	1,308	1.43 (1.25–1.65)	67.10	<0.01	
>500	3	18,607	1.28 (1.22–1.33)	81.30	<0.01	0.13
**Number of study center**						
Single-center	6	7,361	1.37 (1.29–1.45)	70.09	<0.01	
Multi-center	3	12,554	1.21 (1.13–1.26)	79.50	<0.01	<0.01
**Recipient MELD score**						
< =15	3	530	1.28 (1.02–1.61)	41.50	0.10	
>15	2	904	1.23 (1.05–1.44)	46.10	0.10	0.40
**Cold ischemia time (min)**						
< =350	3	447	0.97 (0.76–1.23)	0	0.80	
>350	4	7,417	1.35 (1.27–1.43)	63.00	<0.01	0.01
**Warm ischemia time (min)**						
< =50	2	342	1.18 (0.89–1.56)	50.30	0.09	
>50	2	539	1.15 (0.92–1.43)	43.60	0.13	0.88
**Gender of recipient (male%)**						
< =70	3	7,027	1.32 (1.24–1.40)	73.90	<0.01	
>70	3	811	1.35 (1.14–1.60)	21.50	0.25	0.78
**Gender of donor (male%)**						
< =60	3	1,013	1.38 (1.18–1.60)	34.64	0.12	
>60	2	421	0.98 (0.77–1.25)	0.00	0.61	0.02
**Recipient age (y)**						
< =52	2	904	1.23 (1.05–1.45)	46.10	0.10	
>52	3	530	1.28 (1.02–1.61)	41.50	0.10	0.78
**Year for liver transplantation**						
2001–2006	8	29,702	1.29 (1.24–1.35)	81.60	<0.01	
2007–2012	3	530	1.28 (1.02–1.61)	41.50	0.10	0.95
**Recipient etiology**						
No-hepatitis	2	7,524	0.91 (0.82–1.02)	66.40	0.01	
CHB	1	967	1.52 (1.26–1.84)	0.00	0.59	
CHC	3	9,964	1.38 (1.31–1.45)	87.60	<0.01	<0.01
**Region**						
European	5	1,083	1.25 (1.10–1.42)	39.00	0.07	
USA	4	18,481	1.30 (1.24–1.35)	83.20	<0.01	0.60
**PNF**						
**Sample size**						
< =300	2	460	0.87 (0.45–1.67)	0	0.94	
>300	2	904	1.12 (0.67–1.87)	4.00	0.38	0.54
**Number of study center**						
Single-center	2	460	0.87 (0.45–1.67)	0	0.94	
Multi-center	2	904	1.12 (0.67–1.87)	4.00	0.38	0.54
**Recipient MELD score**						
< =15	2	188	0.87 (0.45–1.67)	0	0.94	
>15	2	904	1.12 (0.67–1.87)	4.00	0.38	0.54
**Cold ischemic time (min)**						
< =400	2	539	0.70 (0.29–1.69)	0	0.53	
>400	2	825	1.12 (0.71–1.77)	0	0.88	0.35
**Warm ischemic time (min)**						
< =50	1	272	0.76 (0.33–1.75)	0	0.97	
>50	2	569	0.70 (0.29–1.69)	0	0.53	0.90
**Gender of recipient (male%)**						
< =70	2	623	0.60 (0.28–1.26)	0	0.82	
>70	2	741	1.27 (0.78–2.05)	0	0.91	0.10
**Gender of donor (male%)**						
< =60	1	188	1.01 (0.65–1.56)	0	0.68	
>60	3	1,176	1.07 (0.37–3.08)	0	0.63	0.91
**Recipient age**						
< =52	2	904	1.12 (0.67–1.87)	4.00	0.38	
>52	2	460	0.87 (0.45–1.67)	0	0.94	0.54
**Year for liver transplantation**						
2003–2006	2	954	1.12 (0.67–1.87)	4	0.38	
2007–2009	2	460	0.87 (0.45–1.67)	0	0.94	0.54
**Re-transplantation**						
**Sample size**						
< =200	1	188	1.75 (0.66–4.64)	17.20	0.30	
>200	2	623	0.96 (0.60–1.52)	37.90	0.17	0.27
**Number of study center**						
Single-center	2	460	1.53 (0.92–2.52)	0	0.60	
Multi-center	1	351	0.47 (0.22–1.01)	0	0.88	0.01
**Recipient MELD score**						
<15	1	272	1.45 (0.81–2.61)	0	0.57	
≥15	2	539	0.77 (0.42–1.41)	40.70	0.15	0.14
**Cold ischemic time (min)**						
< =400	2	539	0.77 (0.42–1.41)	40.70	0.15	
>400	1	272	1.45 (0.81–2.61)	0	0.57	0.14
**Warm ischemic time (min)**						
< =50	2	539	0.77 (0.42–1.41)	40.70	0.15	
>50	1	272	1.45 (0.81–2.61)	0	0.57	0.14
**Gender of recipient (male%)**						
< =70	2	623	0.96 (0.60–1.52)	37.90	0.17	
>70	1	188	1.75 (0.66–4.64)	17.20	0.30	0.27
**Gender of donor (male%)**						
< =60	2	460	1.53 (0.92–2.52)	0	0.60	
>60	1	351	0.47 (0.22–1.01)	0	0.88	0.01
**Recipient age**						
< =52	1	351	0.47 (0.22–1.01)	0	0.88	
>52	2	460	1.53 (0.92–2.52)	0	0.60	0.01
**Year for liver transplantation**						
2003	1	351	0.47 (0.22–1.01)	0	0.88	
2007–2009;	2	460	1.53 (0.92–2.52)	0	0.60	0.01

*PNF, primary non-function; CHB, chronic hepatitis B; CHC, chronic hepatitis C; RR, relative risk; MELD, model of end-stage liver disease*.

a *P-value represented heterogeneity in subgroup*.

b *P-value represented heterogeneity between subgroups*.

### Meta-Regression

The effects of potential confounders on aging-donor-related outcomes were analyzed by meta-regression (Table 6). Sample size and etiology of LT had significant impacts on donor-age-related organ loss (both *P* < 0.05). Studies with a larger sample size had a lower risk of GF (RR = 0.96, *P* < 0.05) (Supplementary Figure 7A). In contrast, studies including CHB/CHC recipients had higher risks of GF in relation to increments in donor age (RR = 1.57 for CHB; RR = 1.55 for CHC, compared with non-viral hepatitis patients, *P* < 0.05) (Table 6, Supplementary Figure 7B). No covariate significantly affected the impact of donor age on patient mortality or PNF incidence. However, type of study center (multi- vs. single-center) significantly affected the impact of donor age on re-transplantation, with multicenter studies having lower risks of re-transplantation (RR = 0.31; *P* < 0.05) (Supplementary Figure 8).

**Table 6:** Meta regression on donor age related risk of post-transplant outcomes in dose response model.

	**Number of studies**	**Number of patients**	**RR (95%CI)**	***I*^**2**^ (%)**	***P* for significance^**a**^**
**Patient mortality**					
Sample size	6	3,240	0.99 (0.95–1.04)	42.85	0.77
Study center (multi-center vs. Single-center)	6	3,240	1.10 (0.87–1.38)	40.32	0.40
Recipient MELD score	4	1,364	0.99 (0.85–1.15)	43.31	0.87
Cold ischemic time (min)	4	1,364	1.00 (1.00–1.00)	41.70	0.85
Warm ischemic time (min)	3	811	1.03 (0.98–1.08)	42.68	0.20
Gender of recipient (male%)	4	1,364	0.98 (0.02–60.24)	43.40	0.99
Gender of donor (male%)	4	1,364	0.16 (0.00–5.582)	36.13	0.29
Year for liver transplantation	6	3,240	1.01 (0.98–1.03)	41.98	0.52
Recipient age (y)	4	1,092	1.12 (0.98–1.28)	22.46	0.08
Study region (America vs. European)	6	3,240	1.00 (0.68–1.47)	42.57	0.99
**Graft failure**					
Sample size	9	19,915	0.97 (0.95–0.99)	70.57	<0.01
Study center (multi-center vs. Single-center)	9	19,915	0.82 (0.64–1.05)	76.65	0.11
Recipient MELD score	5	1,434	1.00 (0.90–1.10)	43.67	0.93
Cold ischemic time (min)	7	7,864	1.00 (0.99–1.00)	42.28	0.08
Warm ischemic time (min)	4	881	1.01 (0.99–1.03)	46.61	0.47
Gender of recipient (male%)	7	15,759	0.23 (0.02–2.23)	50.41	0.19
Gender of donor (male%)	5	1,434	0.27 (0.04–2.07)	35.65	0.19
Recipient age (y)	5	1,434	0.99 (0.91–1.08)	43.72	0.85
Year for liver transplantation	9	19,915	0.99 (0.95–1.03)	78.84	0.59
Recipient etiology	6	18,455		73.03	0.02
CHB vs. No-hepatitis			1.57 (1.05–2.37)		0.03
CHC vs. No-hepatitis			1.55 (1.13–2.13)		0.01
Study region (America vs. European)	9	19,915	1.02 (0.77–1.35)	78.74	0.90
**PNF**					
Sample size	4	1,092	2.21 (0.50–9.85)	0	0.26
Study center (multi-center vs. Single-center)	4	1,092	1.30 (0.50–3.39)	0	0.56
Recipient MELD score	4	1,092	1.12 (0.90–1.40)	0	0.26
Cold ischemic time (min)	4	1,092	1.00 (0.99–1.01)	0	0.92
Warm ischemic time (min)	3	811	1.01 (0.94–1.08)	0	0.87
Gender of recipient (male%)	4	1,092	2.01 (0–3587.44)	0	0.84
Gender of donor (male%)	4	1,092	0.91 (0–5116.04)	0	0.98
Recipient age (y)	4	1,092	1.03 (0.78–1.38)	0	0.79
Year for liver transplantation	4	1,092	1.12 (0.80–1.58)	0	0.47
**Re-transplantation**					
Sample size	3	811	0.98 (0.94–1.03)	30.90	0.39
Study center (multi-center vs. single center)	3	811	0.31 (0.10–0.96)	0.00	0.04
Recipient MELD score	3	811	0.67 (0.42–1.07)	0.00	0.08
Cold ischemic time (min)	3	811	1.00 (0.99–1.01)	9.70	0.13
Warm ischemic time (min)	3	811	0.98 (0.92–1.05)	33.95	0.59
Gender of recipient (male%)	3	811	0.01 (0–1.69)	0	0.06
Gender of donor (male%)	3	811	0.12 (0–299.80)	37.98	0.53
Recipient age (y)	3	811	1.18 (0.80–1.76)	0	0.34
Year for liver transplantation	3	811	1.27 (0.98–1.62)	0.00	0.05

*PNF, primary non-function; CHB, chronic hepatitis B; CHC, chronic hepatitis C; RR, relative risk; MELD, model of end-stage liver disease*.

a *P-value represented the significance about coefficient vector (b)*.

### Influence Analysis

Sensitivity analysis was performed to visualize the impact of each study on the overall risks. The trend of pooled categorical risk was consistent. No study had any apparent influence on the overall results (Supplementary Figure 9). Regarding the continuous results however, omitting the study by Oscar et al. had a marked effect on patient mortality (Supplementary Figure 11) (24).

### Publication Bias

No significant publication bias was observed in relation to continuous risk assessment (all *P* > 0.05) (Supplementary Figure 12). However, for categorical assessment, there was significant publication bias regarding the risk of re-transplantation in the older-donor age group (Table 1, Supplementary Figure 10D).

## Discussion

To the best of our knowledge, this is the first study to focus on the association between donor age and prognosis on recipient outcomes after LT, based on systematic analysis of quantitative data from the published literature. Analysis of 11 studies, including 30,691 cadaveric LT donors, showed that: (1) using grafts from older donors significantly increased the pooled risks of patient mortality and GF by about 114 and 66%, respectively (both *P* < 0.05); (2) older donors had a significant effect on post-transplant prognosis, showing a linear trend, with increments in patient death and GF of about 5% and 12% per 10 year increase in donor age (both *P* < 0.05); (3) the use of older grafts had no significant effect on the incidence of PNF or re-transplantation, as short-term post-transplant complications (both *P* > 0.05), but use of female donors, prolonged CIT, and primary viral hepatitis might amplify the effects of graft age on GF (all *P* < 0.05); and (4) compared with the previous empirical definition of 60 years (40), the safety cutoff for donor age should be lowered to around 43 years to ensure a comparable prognosis with cases treated using younger grafts.

In accordance with previous study (2), our results confirmed that advanced donor age had adverse impacts on patient post-transplant prognosis. In terms of overall patient mortality, the risk peaked within 1 year after LT, represented by higher categorical and continuous RRs. Consistently, the risk of GF was increased in patients treated with older grafts, but the peak RR was delayed to the 3rd year after LT. A 10-year increment in donor age increased the risks of patient and graft mortality by about 5 and 12%, respectively, and increased donor age appeared to affect post-transplant outcomes in a linear manner. However, donor age had little impact on short-term post-transplant complications, such as PNF and re-transplantation. Consistent with our study, two studies found no significant relationship between donor age and PNF or re-transplantation occurrence in LT cases from UNOS (41, 42). As we have known, regenerative capacity of the liver is generally declined with age, with reflection on lower proliferation in livers from older donors (3, 43–46). Grafts from older donors are more susceptible to IRI with inferior post-transplant prognosis (47). However, in the early stage after LT, the overall graft functions from aging donors remained well-preserved due to their effective counterbalance by large, functional reserve (48). And the mechanism referred above might partially explain why donor aging had insignificant effects on short-term complications after LT (48). Followed with the extension on post-transplant duration, this reserved liver function is exhausted, and the adverse impacts of donor age on prognosis would become more and more significant.

The safety cutoff for donor age for LT has not previously been defined (3), and cutoffs have been decided empirically, with no systematic evidence (27). In the current study, we determined the safety threshold for donor age based on the risks of patient death and GF. Unlike the relatively higher cutoff for patient death, the effects of donor age on GF suggested that the threshold should be lowered to 43 years, to guarantee graft quality. More attentions should be paid to prevent the possible GF in patients received grafts from elder donors. A similar conclusion was reached in a multicenter study from the US, which identified increased donor age (>40 years) as a strong risk factor for GF (6). Increasing numbers of patients are currently on waiting lists for LT due to organ shortages (49), potentially leading surgeons to consider marginally suitable grafts from older donors, given careful donor and recipient matching (50). Some surgeons suggest that livers from donors aged >60 years should routinely be discarded (51), while others found that similar outcomes could be achieved with older allografts, with careful donor/recipient selection, limited CIT, and lower MELD score (52). Importantly, the mortality of patients waiting for LT can't be ignored (53) and the use of extended-criteria donors (ECD) might help to reduce waiting list mortality. The use of grafts from older donors may thus help to solve the organ shortage to some extent (54), though the lower quality of ECD grafts is associated with increased risks. The current study systematically evaluated the effects of grafts from elder donors on post-transplant outcomes, and these results might help clinicians to make better decisions to balance the risks and benefits when using elder grafts for reducing the waiting list mortality. The safety threshold at 43 years is an early warning for clinicians so that they can better predict the risk of prognosis and provide timely interventions to prevent adverse outcomes by other measurements to guarantee the LT quality.

Despite the above results, many studies also reported potential confounders of the association between donor age and post-transplant outcomes. Grazi et al. found that lower recipient MELD score and shorter CIT helped to attenuate the burden associated with aged donor livers (55), while another study found that older organs were associated with a better prognosis in patients with malignancy and stable liver function compared with those without these conditions (56). If clinicians can avoid factors such as increased surgical ischemia time and recipients with higher MELD scores, the results of LT using older donors may be similar to those with younger donors (3). Consistent with prior studies, our results revealed that older donor age was associated with a significant risk of GF in cases with relatively longer CIT. However, the increased risk of GF associated with older donors was not significant in studies of patients without viral hepatitis. Intriguingly, the donor-age-related risk was also reduced in studies with a larger sample size, with about a 3% reduction in risk per 100 increment in LT cases. This may suggest that increasing experience of LT reduced the aging-organ-related risk in a gentle but steady trend. Our results indicated that the adverse effects from elder organs could be offset by strict recipient selection and improved surgical technology. Sensitivity analysis showed that one study (24) affected the pooled results because of its lower RR of patient death, possibly because of a lower proportion of older donors in this study.

Elder donors may suffer more frequently from diabetes and obesity, which might affect the relationship between donor age and post-transplant outcomes. The pooled BMI and prevalence of diabetes were higher in the older donors compared to youngers (both *P* < 0.05).

We suspect obesity and diabetes in elder donors might be responsible for worse outcomes in patients received older organs. Further studies for exact estimation on association between aging organ and post-transplant prognosis by adjustment of donors' metabolic derangement are needed to fully confirm our speculation.

The current study had some limitations. First, some studies were performed in single center with only 70 cases (20), and the results may therefore not be representative, due to the limited number of cases. Second, few studies provided information on occurrence of PNF and re-transplantation, which might also limit the accurate estimation of relevant results. And the lower occurrence for inferior events in short-term follow-up duration might also cause bias of pooled results. Otherwise, differences on definitions of PNF across studies might also affect the pooled results (Supplementary Table 4). Third, the safety of the threshold donor age could not be assessed for the limited data available for 2 and 3-year patient mortality and 5-year GF. Fourth, differences in recipient ages between subgroups according to donor age might also have contributed to selection bias in the pooled results. Finally, we wanted to emphasize that the exact estimation on the effect of comorbidities such as obesity and diabetes from donors on the relationship between donor age and post-transplant outcomes would require that all data from primary studies should be adjusted for the same factors. It's impossible for us to do it, because this is the intrinsic limitation of meta-analysis (57).

In conclusion, donor age might have adverse dose-response effects on patient death and GF following LT. Factors including primary viral hepatitis in recipients and extended CIT might aggravate the risk of GF associated with increased donor age. The safety for threshold donor age can be lowered to 43 years, as early warning for guarantee of satisfactory post-transplant outcomes. Further studies are needed to clarify the mechanisms underlying this association.

## Data Availability Statement

The original contributions generated for the study are included in the article/Supplementary Material, further inquiries can be directed to the corresponding author/s.

## Ethics Statement

This study was performed in accordance with the Declaration of Helsinki and approved by the ethical board of First Affiliated Hospital, School of Medicine, Zhejiang University.

## Author Contributions

ZL, WW, and SZ conceived and designed the study. WW, JQ, SQ, JX, and ZL extracted information, analyzed the data, and wrote the manuscript. WW, LZho, LZhu, LG, and SZ reviewed the manuscript. All authors approved the final manuscript for submission.

## Conflict of Interest

The authors declare that the research was conducted in the absence of any commercial or financial relationships that could be construed as a potential conflict of interest.

## Supplementary Material

Supplementary Figure 1 Graphical abstract of the study.

Supplementary Figure 2 Comparisons on HAT and ITBL occurrence in groups categorized by donor age. HAT, hepatic artery thrombosis; ITBL, ischemic type biliary lesion.

Supplementary Figure 3 Forest plot on pooled SMD of advanced donor age on length of hospitalization. **(A)** Pooled SMD of advanced donor age on length of hospital after LT. **(B)** Pooled SMD of advanced donor age on length of ICU stay. SMD, standardized mean differences; ICU, intensive care unit.

Supplementary Figure 4 Forest plot on pooled SMD of advanced donor age on BMI. SMD, standardized mean differences; BMI, body mass index.

Supplementary Figure 5 Comparisons of diabetes prevalence in groups categorized by donor age.

Supplementary Figure 6 Dose response analysis on risk of advanced donor age on LT related complications. **(A)** Dose-response risk of advanced donor age on risk of PNF occurrence. **(B)** Dose-response risk of advanced donor age on risk of re-transplantation incidence. LT, liver transplantation; PNF, primary non-function.

Supplementary Figure 7 Meta-regression on impact of potential confounders on aging graft related GF risk. **(A)** Meta regression on impact of sample size on donor age related GF. **(B)** Meta regression on impact of recipients' viral hepatitis status on donor age related GF. GF, graft failure.

Supplementary Figure 8 Meta regression revealed the impact of center number on donor age related risk of re-transplantation.

Supplementary Figure 9 Sensitivity analyses on pooled categorical risk of post-transplant outcomes by stepwise omitting each study at a time. Risk was evaluated based on comparison between elder and younger donors. **(A)** Re-evaluation of donor age related GF risk after omitting each study. **(B)** Re-evaluation of donor age related risk of patient mortality after omitting each study. **(C)** Re-evaluation of donor age related risk of PNF after omitting each study. **(D)** Re-evaluation of donor age related risk of re-transplantation after omitting each study. GF, graft failure; PNF, primary non-function.

Supplementary Figure 10 Funnel plot analysis on publication bias of categorical risks of aging on post-transplant outcomes. Risk of post-transplant outcomes was compared in recipients using grafts from elder and younger donors. **(A)** Begg's funnel plot on publication bias of risks on donor age related GF; *P* for egger's test = 0.66. **(B)** Begg's funnel plot on publication bias of risks on donor age related patient mortality; *P* for egger's test = 0.89. **(C)** Begg's funnel plot on publication bias of risks on donor age related PNF; *P* for egger's test = 0.05. **(D)** Begg's funnel plot on publication bias of risks on donor age related re-transplantation; *P* for egger's test = 0.01. HR, hazard ratio; GF, graft failure; RR, relative risk; PNF, primary non-function.

Supplementary Figure 11 Sensitivity analysis on continuous aging related risk by stepwise omitting one study at a time. Risk was evaluated followed per 10-year of donor age increment. **(A)** Sensitivity analysis on continuous aging related GF risk by stepwise omitting one study at a time. **(B)** Sensitivity analysis on continuous aging related risk of patient death by stepwise omitting one study at a time. **(C)** Sensitivity analysis on continuous aging related PNF risk by stepwise omitting one study at a time. **(D)** Sensitivity analysis on continuous aging related risk of re-transplantation by stepwise omitting one study at a time. GF, graft failure.

Supplementary Figure 12 Funnel plot analysis on publication bias of continuous risks of aging on post-transplant outcomes. Risk was evaluated followed per 10-year of donor age increment. **(A)** Begg's funnel plot on publication bias of continuous risks of GF; *P* for egger's test = 0.98. **(B)** Begg's funnel plot on publication bias of continuous risks of patient death; *P* for egger's test = 0.05. **(C)** Begg's funnel plot on publication bias of continuous risks of PNF; *P* for egger's test = 0.13. **(D)** Begg's funnel plot on publication bias of continuous risks of re-transplantation; *P* for egger's test = 0.14. GF, graft failure; RR, relative risk; PNF, primary non-function.

## Abbreviations

BMI: body mass index
CIT: cold ischemia time
DCD: donation after cardiac death
DBD: donation after brain death
GF: graft failure
GLS: generalized least-squares
HCV: hepatitis C virus
HBV: hepatitis B virus
HCC: hepatocellular carcinoma
HAT: hepatic artery thrombosis
HR: hazard ratio
IRI: ischemia reperfusion injury
IQR: inter-quartile range
ICU: intensive care unit
ITBL: ischemic type biliary lesion
LT: Liver transplantation
MELD: model of end-stage liver disease
NOS: Newcastle-Ottawa Scale
NAS: non-anastomotic biliary strictures
PNF: primary non-function
RR: relative risk
SD: standard deviation
SMD: standardized mean difference
UNOS: United Network for Organ Sharing
WIT: warm ischemia time.
