# Supplementary material for: Systematic Assessment of Safety Threshold for Donor Age in Cadaveric Liver Transplantation
Source: Front Med (Lausanne). 2021 Mar 4;8:596552. doi: 10.3389/fmed.2021.596552 (PMC7969668; doi:10.3389/fmed.2021.596552)
Supplement: Supplementary file 1 [file Data_Sheet_1.docx]

Table S1 Literature search strategy across different databases

| ISI-web of science | Indexes=SCI-EXPANDED, SSCI, A&HCI, CPCI-S, CPCI-SSH, ESCI, CCR-EXPANDED, IC Timespan=1980-2018 |  |
| --- | --- | --- |
| #1 | (TS= (donor age)) AND DOCUMENT TYPES:(Article) | 32069 |
| #2 | (TS= (aging donor)) AND DOCUMENT TYPES:(Article) | 32069 |
| #3 | (TS= (aging)) AND DOCUMENT TYPES:(Article) | 2600819 |
| #4 | \| (TS=(age)) AND DOCUMENT TYPES:(Article) \| \| --- \| | 2600819 |
| #5 | (TS= (old donor)) AND DOCUMENT TYPES:(Article) | 14132 |
| #6 | (TS= (older donor)) AND DOCUMENT TYPES:(Article) | 14088 |
| #7 | #1 OR #2 OR #3 OR #4 OR #5 OR #6 | 2608147 |
| #8 | (TS= (liver transplantation)) AND DOCUMENT TYPES: (Article) | 66473 |
| #9 | (TS= (mortality)) AND DOCUMENT TYPES: (Article) | 759074 |
| #10 | (TS= (survival)) AND DOCUMENT TYPES: (Article) | 961242 |
| #11 | #9 OR #10 | 1562360 |
| #12 | #7 AND #8 AND #11 | 6090 |
| Embase |  |  |
| #1 | 'donor age' AND [article]/lim | 4002 |
| #2 | 'aging donor' AND [article]/lim | 13 |
| #3 | 'aging' AND [article]/lim | 528232 |
| #4 | age AND [article]/lim | 2390130 |
| #5 | 'old donor' AND [article]/lim | 323 |
| #6 | 'older donor' AND [article]/lim | 369 |
| #7 | #1 OR #2 OR #3 OR #4 OR #5 OR #6 | 2744352 |
| #8 | 'liver transplantation' AND [article]/lim | 55373 |
| #9 | 'mortality' AND [article]/lim | 816513 |
| #10 | 'survival' AND [article]/lim | 980033 |
| #11 | #9 OR #10 | 1608966 |
| #12 | #7 AND #8 AND #11 | 5011 |
| PubMed |  |  |
| #1 | Search donor age Filters: Full text | 30972 |
| #2 | Search aging donor Filters: Full text | 5126 |
| #3 | Search aging Filters: Full text | 397474 |
| #4 | Search age Filters: Full text | 2098079 |
| #5 | Search old donor Filters: Full text | 10949 |
| #6 | Search older donor Filters: Full text | 5254 |
| #7 | ((((donor age OR aging donor) OR age) OR old donor) OR older donor) OR aging) Filters: Full text | 2322480 |
| #8 | Search liver transplantation Filters: Full text | 89787 |
| #9 | Search mortality Filters: Full text | 1015313 |
| #10 | Search survival Filters: Full text | 1741822 |
| #11 | Search (mortality) OR survival Filters: Full text | 1797110 |
| #12 | **(((((donor age OR aging donor) OR age) OR old donor)** OR older donor) **OR aging) AND (liver transplantation) AND ((survival) OR mortality)** Filters: **Full text** | 6945 |

Table S2 Newcastle-Ottawa scale (NOS) scale for quality assessment to enrolled studies

|  | Faber et al, 2001, Germany | Lai et al.,2011, Italy | Detry et al, 2014, Belgium | Machicao et al., 2004, USA | Russo et al.,2004, USA | Lakea et al., 2005, USA | Carlos et al, 2013, Spain | Matteo et al, 2008, Italy | Katherine et al, 1995, USA | Wesley et al, 1991, USA | Oscar et al, 2005, Spain | David et al,2006, European |
| --- | --- | --- | --- | --- | --- | --- | --- | --- | --- | --- | --- | --- |
| **Selection** |  |  |  |  |  |  |  |  |  |  |  |  |
| Representativeness of the cohort using older donor group | 1 | 1 | 1 | 1 | 1 | 1 | 1 | 1 | 1 | 1 | 1 | 0 |
| Selection of the cohort using younger donor group | 1 | 1 | 1 | 1 | 1 | 1 | 1 | 1 | 1 | 1 | 1 | 0 |
| Ascertainment of older donor | 1 | 1 | 1 | 1 | 1 | 1 | 1 | 1 | 1 | 1 | 1 | 1 |
| Demonstration that inferior outcomes were excluded or distinguished at start of study | 1 | 1 | 1 | 1 | 1 | 1 | 1 | 1 | 1 | 1 | 1 | 1 |
| **Comparison** |  |  |  |  |  |  |  |  |  |  |  |  |
| Controls for recipient MELD score | 1 | 1 | 1 | 1 | 1 | 0 | 1 | 1 | 1 | 0 | 1 | 0 |
| Controls for cold ischemic time | 1 | 1 | 1 | 1 | 1 | 0 | 1 | 0 | 1 | 0 | 1 | 0 |
| **Outcome** |  |  |  |  |  |  |  |  |  |  |  |  |
| Assessment of post-operative outcomes | 1 | 1 | 1 | 1 | 1 | 1 | 1 | 1 | 1 | 1 | 1 | 1 |
| Long enough follow-up to evaluate outcomes | 0 | 1 | 1 | 1 | 1 | 1 | 1 | 1 | 0 | 0 | 1 | 1 |
| Adequate follow up of cohorts | 1 | 0 | 0 | 0 | 1 | 1 | 1 | 1 | 0 | 1 | 1 | 0 |
| Total (9 as maximum) | 8 | 8 | 8 | 8 | 9 | 7 | 9 | 8 | 7 | 6 | 9 | 4 |

MELD, model for end-stage liver disease.

Table S3 Major indicators observed in enrolled studies

| Author, year, country | Patients’ Survival | | | | | | Organ Failure | | | | | | Complications | | | |  |
| --- | --- | --- | --- | --- | --- | --- | --- | --- | --- | --- | --- | --- | --- | --- | --- | --- | --- |
|  | 90 day | 180 day | 1 year | 2 year | 3 year | 5 year | 90 day | 180 day | 1 year | 2 year | 3 year | 5 year | Re-transplantation | PNF | Hepatic artery thrombosis | Ischemic biliary lesion | Length of hospitalization |
| Faber et al.,2001, Germany | Yes | Yes | Yes | NA | NA | NA | Yes | Yes | Yes | NA | NA | NA | Yes | Yes | NA | Yes | In ward / ICU |
| Quirino et al.,2011, Italy | yes | Yes | Yes | Yes | Yes | Yes | Yes | Yes | Yes | Yes | Yes | Yes | yes | yes | NA | NA | NA |
| Detry et al., 2014, Belgium | NA | NA | NA | NA | NA | NA | Yes | Yes | Yes | Yes | Yes | NA | NA | NA | NA | NA | NA |
| Victor et al., 2004, USA | NA | NA | NA | NA | NA | NA | Yes | Yes | Yes | Yes | Yes | NA | NA | NA | NA | NA | NA |
| Mark et al., 2004, USA | NA | NA | NA | NA | NA | NA | Yes | Yes | Yes | Yes | Na | NA | NA | NA | NA | NA | NA |
| John et al., 2005, USA | NA | NA | NA | NA | NA | NA | Yes | Yes | Yes | Yes | Yes | NA | NA | NA | NA | NA | NA |
| Carlos et al.,2013, Spain | Yes | Yes | Yes | Yes | Yes | Yes | Yes | Yes | Yes | Yes | Yes | Yes | Yes | Yes | Yes | NA | In ward / ICU |
| Matteo et al., 2008, Italy | Yes | Yes | Yes | Yes | Yes | Yes | Yes | Yes | Yes | Yes | Yes | Yes | NA | Yes | NA | NA | NA |
| Katherine et al., 1995, USA | NA | Yes | NA | NA | NA | NA | NA | Yes | NA | NA | NA | NA | NA | NA | NA | NA | NA |
| Wesley et al., 1991, USA | Yes | Yes | Yes | NA | NA | NA | NA | Na | NA | NA | NA | NA | NA | NA | NA | NA | NA |
| Oscar et al.,2005, Spain | Yes | Yes | Yes | Yes | Yes | Yes | NA | NA | NA | NA | NA | NA | NA | NA | NA | NA | NA |

ALT, alanine aminotransferase; AST, aspartate aminotransferase; ICU, intensive care unit; Na, not available; PNF, primary non-function.

Table S4 Definition of primary nonfunction in enrolled studies

| Study (author, year, country) | PNF |
| --- | --- |
| Faber et al,2011, Germany | Graft failure in 14 days after liver transplantation. |
| Quirino et al,2011, Italy | Initial no function of the allograft during the first week after liver transplantation. |
| Carlos et al,2013, Spain | GOT>1,500 IU/L, prothrombin rate<60 %, and if the recipient died or required urgent re-transplantation within the first 14 days, having excluded extrahepatic causes. |
| Matteo et al,2008, Italy | A condition of liver failure that occurred in the absence of technical or immunological problems, which led to re-LT or death within the first month after surgery. |

PNF, primary non-function; GOT, glutamic oxaloacetic transaminase; LT, liver transplantation.

Table S5 Comparison on continuous covariates across subgroups categorized by different donor ages

| Item | Comparison | Number of studies | Number of patients | SMD | I*^2^*(%) | p-value for  heterogeneity ^a^ | p-value  for egger's test ^b^ |
| --- | --- | --- | --- | --- | --- | --- | --- |
| **Patient** |  |  |  |  |  |  |  |
| Cold ischemic time | Older vs Younger | 5 | 1434 | -0.02(-0.19-0.15) | 81.3 | <0.01 | 0.05 |
|  | Middle vs Younger |  |  | 0.09(-0.05-0.24) | 0 | 0.45 | 0.75 |
| Warm ischemic time | Older vs Younger | 4 | 881 | -0.12(-0.32-0.07) | 64.10 | 0.04 | 0.16 |
|  | Middle vs Younger |  |  | -0.13(-0.30-0.05) | 86.30 | <0.01 | 0.42 |
| Recipient MELD score | Older vs Younger | 5 | 1434 | 0.05(-0.12-0.22) | 88.30 | <0.01 | 0.13 |
|  | Middle vs Younger |  |  | 0.01(-0.13-0.15) | 81.70 | <0.01 | 0.44 |
| Recipient age | Older vs Younger | 6 | 1460 | 0.25(0.08-0.42) | 51.80 | 0.07 | 0.10 |
|  | Middle vs Younger |  |  | 0.31(0.17-0.45) | 81.50 | <0.01 | 0.22 |

SMD, standardized mean difference; MELD, model for end-stage liver disease.

^a^: P value represented the heterogeneity of pooled results.

^b^: P value represented the publication bias of pooled results.

**Supplementary. PRISMA Checklist**

| **Section/topic** | **#** | **Checklist item** | **Reported on page #** |
| --- | --- | --- | --- |
| **TITLE** | | |  |
| Title | 1 | Identify the report as a systematic review, meta-analysis, or both. | 1 |
| **ABSTRACT** | | |  |
| Structured summary | 2 | Provide a structured summary including, as applicable: background; objectives; data sources; study eligibility criteria, participants, and interventions; study appraisal and synthesis methods; results; limitations; conclusions and implications of key findings; systematic review registration number. | 1-2 |
| **INTRODUCTION** | | |  |
| Rationale | 3 | Describe the rationale for the review in the context of what is already known. | 4-5 |
| Objectives | 4 | Provide an explicit statement of questions being addressed with reference to participants, interventions, comparisons, outcomes, and study design (PICOS). | 4-5 |
| **METHODS** | | |  |
| Protocol and registration | 5 | Indicate if a review protocol exists, if and where it can be accessed (e.g., Web address), and, if available, provide registration information including registration number. | 6 |
| Eligibility criteria | 6 | Specify study characteristics (e.g., PICOS, length of follow-up) and report characteristics (e.g., years considered, language, publication status) used as criteria for eligibility, giving rationale. | 6 |
| Information sources | 7 | Describe all information sources (e.g., databases with dates of coverage, contact with study authors to identify additional studies) in the search and date last searched. | 6 |
| Search | 8 | Present full electronic search strategy for at least one database, including any limits used, such that it could be repeated. | 6 |
| Study selection | 9 | State the process for selecting studies (i.e., screening, eligibility, included in systematic review, and, if applicable, included in the meta-analysis). | 6 |
| Data collection process | 10 | Describe method of data extraction from reports (e.g., piloted forms, independently, in duplicate) and any processes for obtaining and confirming data from investigators. | 7 |
| Data items | 11 | List and define all variables for which data were sought (e.g., PICOS, funding sources) and any assumptions and simplifications made. | 7 |
| Risk of bias in individual studies | 12 | Describe methods used for assessing risk of bias of individual studies (including specification of whether this was done at the study or outcome level), and how this information is to be used in any data synthesis. | 7-8 |
| Summary measures | 13 | State the principal summary measures (e.g., risk ratio, difference in means). | 7-8 |
| Synthesis of results | 14 | Describe the methods of handling data and combining results of studies, if done, including measures of consistency (e.g., I^2^) for each meta-analysis. | 7-8 |

Page 1 of 2

| **Section/topic** | **#** | **Checklist item** | **Reported on page #** |
| --- | --- | --- | --- |
| Risk of bias across studies | 15 | Specify any assessment of risk of bias that may affect the cumulative evidence (e.g., publication bias, selective reporting within studies). | 7-8 |
| Additional analyses | 16 | Describe methods of additional analyses (e.g., sensitivity or subgroup analyses, meta-regression), if done, indicating which were pre-specified. | 7-8 |
| **RESULTS** | | |  |
| Study selection | 17 | Give numbers of studies screened, assessed for eligibility, and included in the review, with reasons for exclusions at each stage, ideally with a flow diagram. | 9 |
| Study characteristics | 18 | For each study, present characteristics for which data were extracted (e.g., study size, PICOS, follow-up period) and provide the citations. | 9 |
| Risk of bias within studies | 19 | Present data on risk of bias of each study and, if available, any outcome level assessment (see item 12). | 10 |
| Results of individual studies | 20 | For all outcomes considered (benefits or harms), present, for each study: (a) simple summary data for each intervention group (b) effect estimates and confidence intervals, ideally with a forest plot. | 10 |
| Synthesis of results | 21 | Present results of each meta-analysis done, including confidence intervals and measures of consistency. | 10-12 |
| Risk of bias across studies | 22 | Present results of any assessment of risk of bias across studies (see Item 15). | 12-13 |
| Additional analysis | 23 | Give results of additional analyses, if done (e.g., sensitivity or subgroup analyses, meta-regression [see Item 16]). | 12-13 |
| **DISCUSSION** | | |  |
| Summary of evidence | 24 | Summarize the main findings including the strength of evidence for each main outcome; consider their relevance to key groups (e.g., healthcare providers, users, and policy makers). | 14-16 |
| Limitations | 25 | Discuss limitations at study and outcome level (e.g., risk of bias), and at review-level (e.g., incomplete retrieval of identified research, reporting bias). | 16-17 |
| Conclusions | 26 | Provide a general interpretation of the results in the context of other evidence, and implications for future research. | 17 |
| **FUNDING** | | |  |
| Funding | 27 | Describe sources of funding for the systematic review and other support (e.g., supply of data); role of funders for the systematic review. | 18 |

*From:*  Moher D, Liberati A, Tetzlaff J, Altman DG, The PRISMA Group (2009). Preferred Reporting Items for Systematic Reviews and Meta-Analyses: The PRISMA Statement. PLoS Med 6(7): e1000097. doi:10.1371/journal.pmed1000097

For more information, visit: **www.prisma-statement.org**.

Page 2 of 2
