# Supplementary figures and images for: Systematic Assessment of Safety Threshold for Donor Age in Cadaveric Liver Transplantation
Source: Front Med (Lausanne). 2021 Mar 4;8:596552. doi: 10.3389/fmed.2021.596552 (PMC7969668; doi:10.3389/fmed.2021.596552)

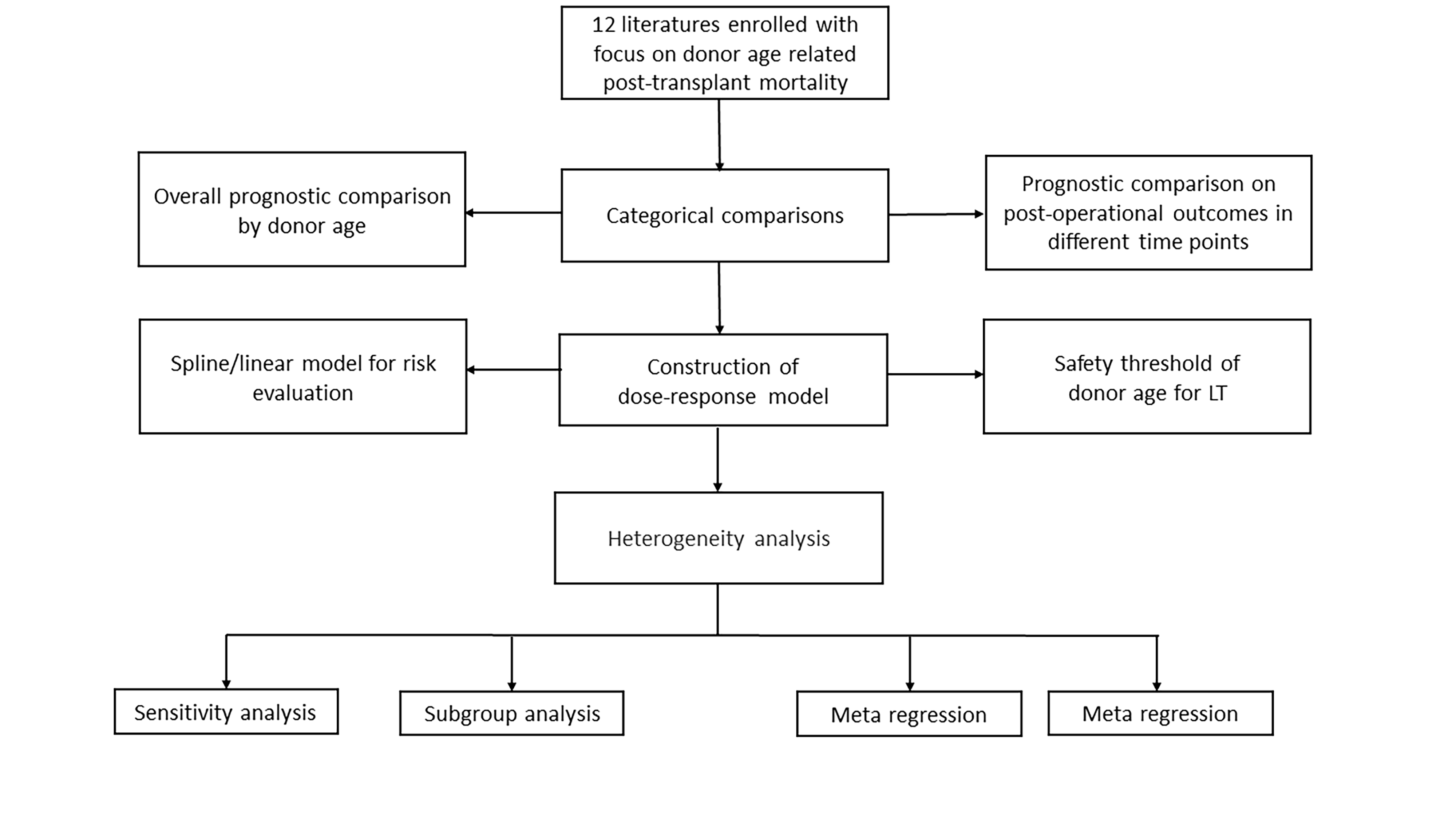

Supplement: Supplementary Figure 1 — Graphical abstract of the study. [file Image_1.TIF]

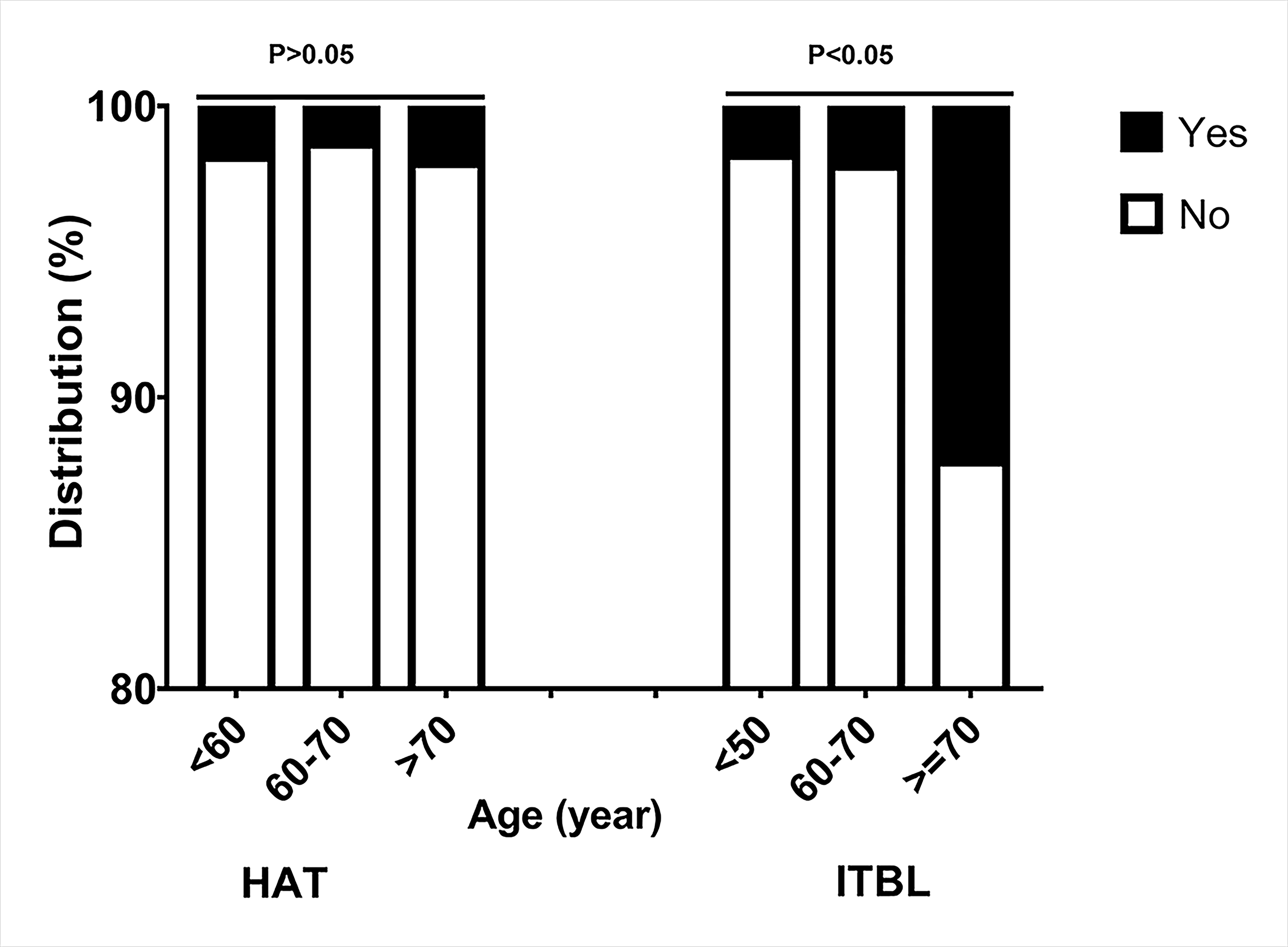

Supplement: Supplementary Figure 2 — Comparisons on HAT and ITBL occurrence in groups categorized by donor age. HAT, hepatic artery thrombosis; ITBL, ischemic type biliary lesion. [file Image_2.TIF]

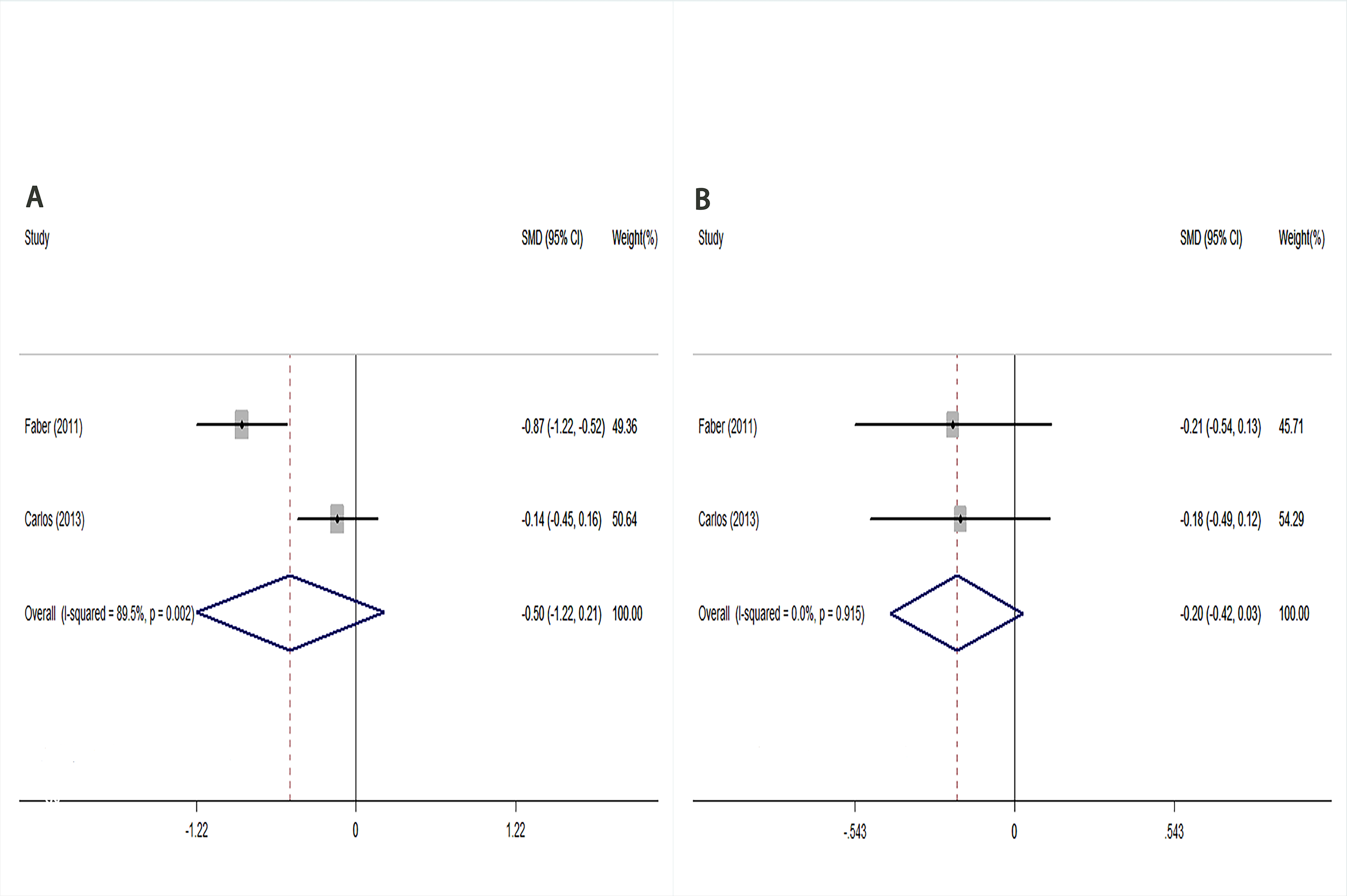

Supplement: Supplementary Figure 3 — Forest plot on pooled SMD of advanced donor age on length of hospitalization. (A) Pooled SMD of advanced donor age on length of hospital after LT. (B) Pooled SMD of advanced donor age on length of ICU stay. SMD, standardized mean differences; ICU, intensive care unit. [file Image_3.TIF]

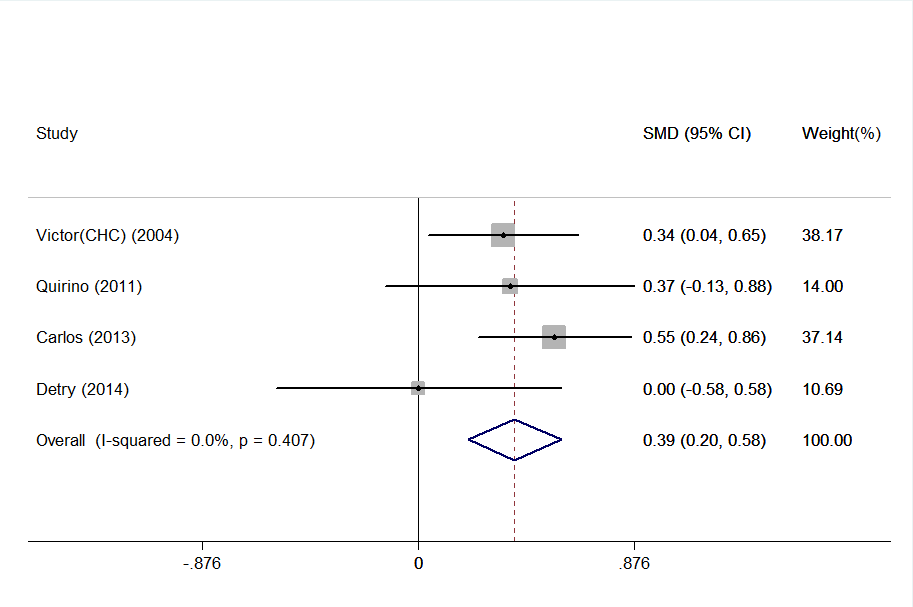

Supplement: Supplementary Figure 4 — Forest plot on pooled SMD of advanced donor age on BMI. SMD, standardized mean differences; BMI, body mass index. [file Image_4.TIF]

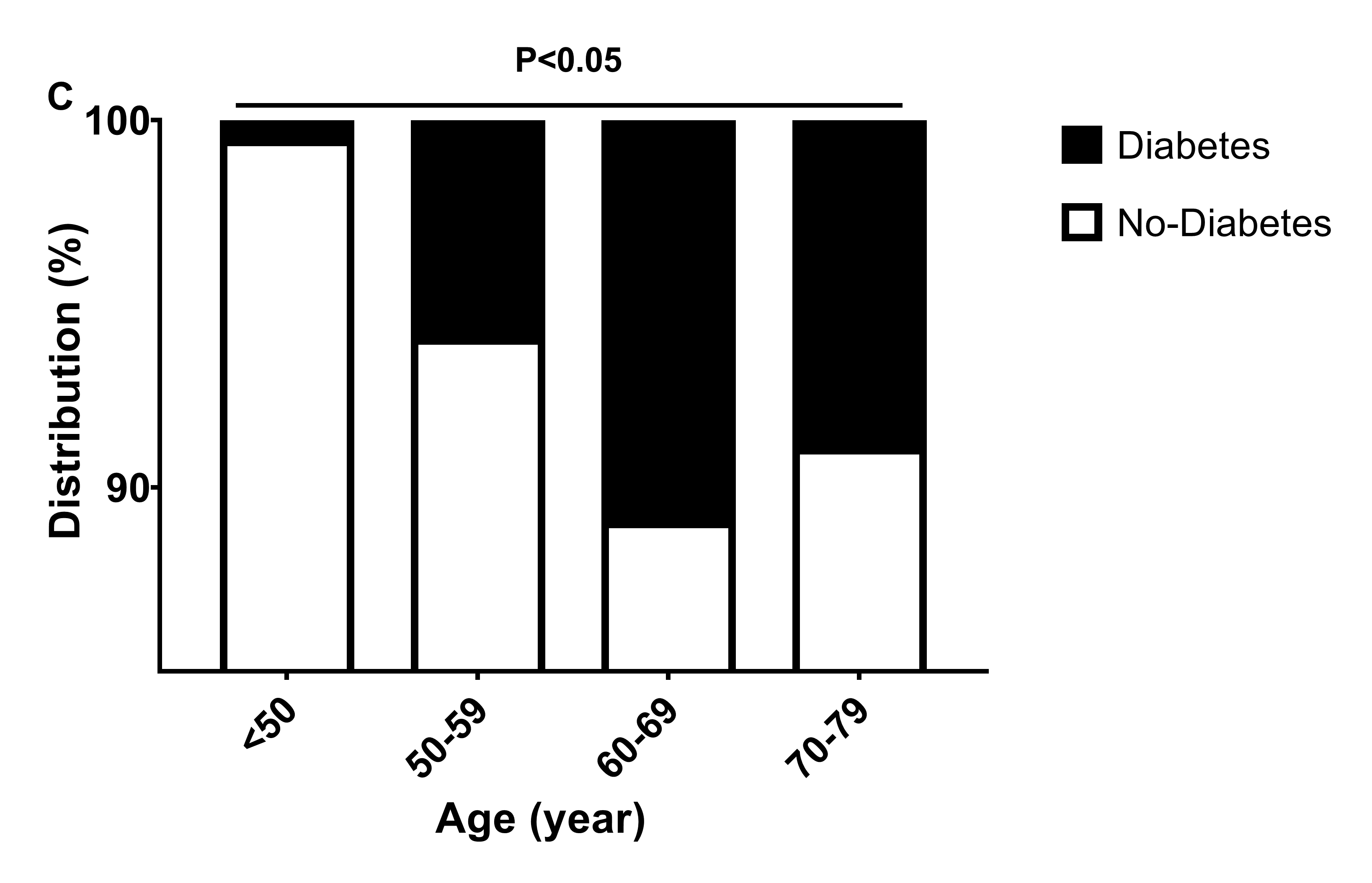

Supplement: Supplementary Figure 5 — Comparisons of diabetes prevalence in groups categorized by donor age. [file Image_5.TIF]

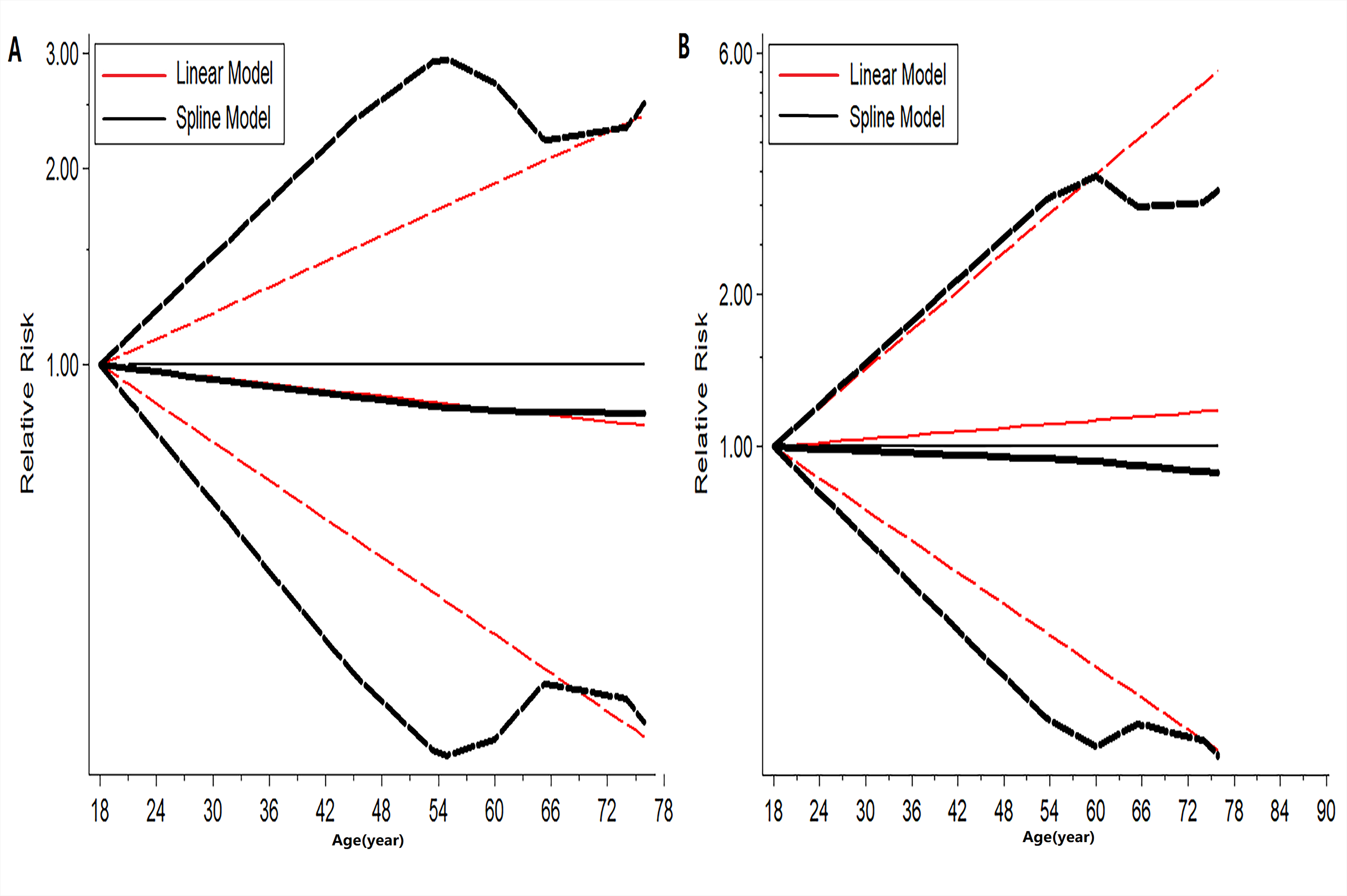

Supplement: Supplementary Figure 6 — Dose response analysis on risk of advanced donor age on LT related complications. (A) Dose-response risk of advanced donor age on risk of PNF occurrence. (B) Dose-response risk of advanced donor age on risk of re-transplantation incidence. LT, liver transplantation; PNF, primary non-function. [file Image_6.TIF]

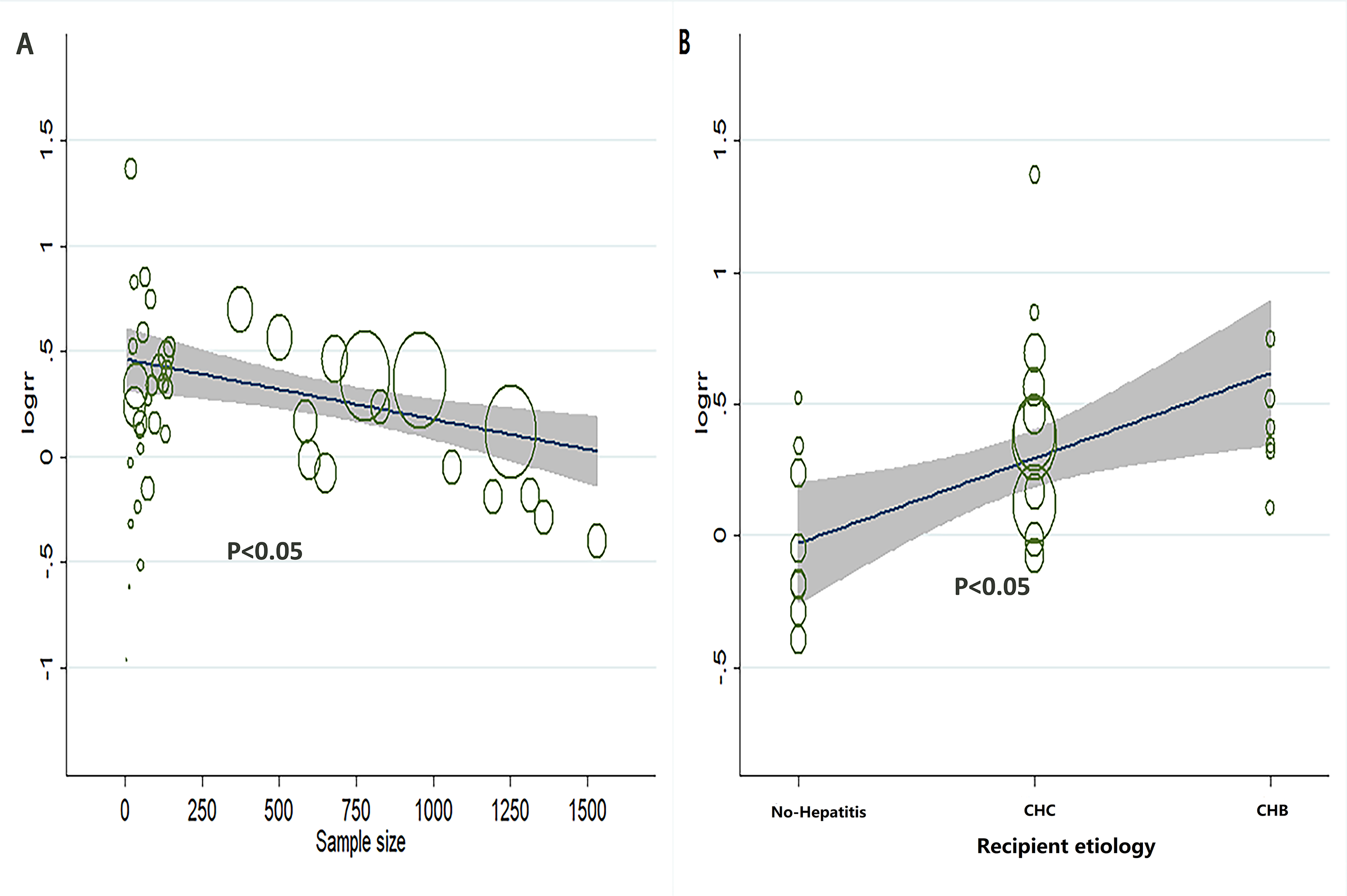

Supplement: Supplementary Figure 7 — Meta-regression on impact of potential confounders on aging graft related GF risk. (A) Meta regression on impact of sample size on donor age related GF. (B) Meta regression on impact of recipients' viral hepatitis status on donor age related GF. GF, graft failure. [file Image_7.TIF]

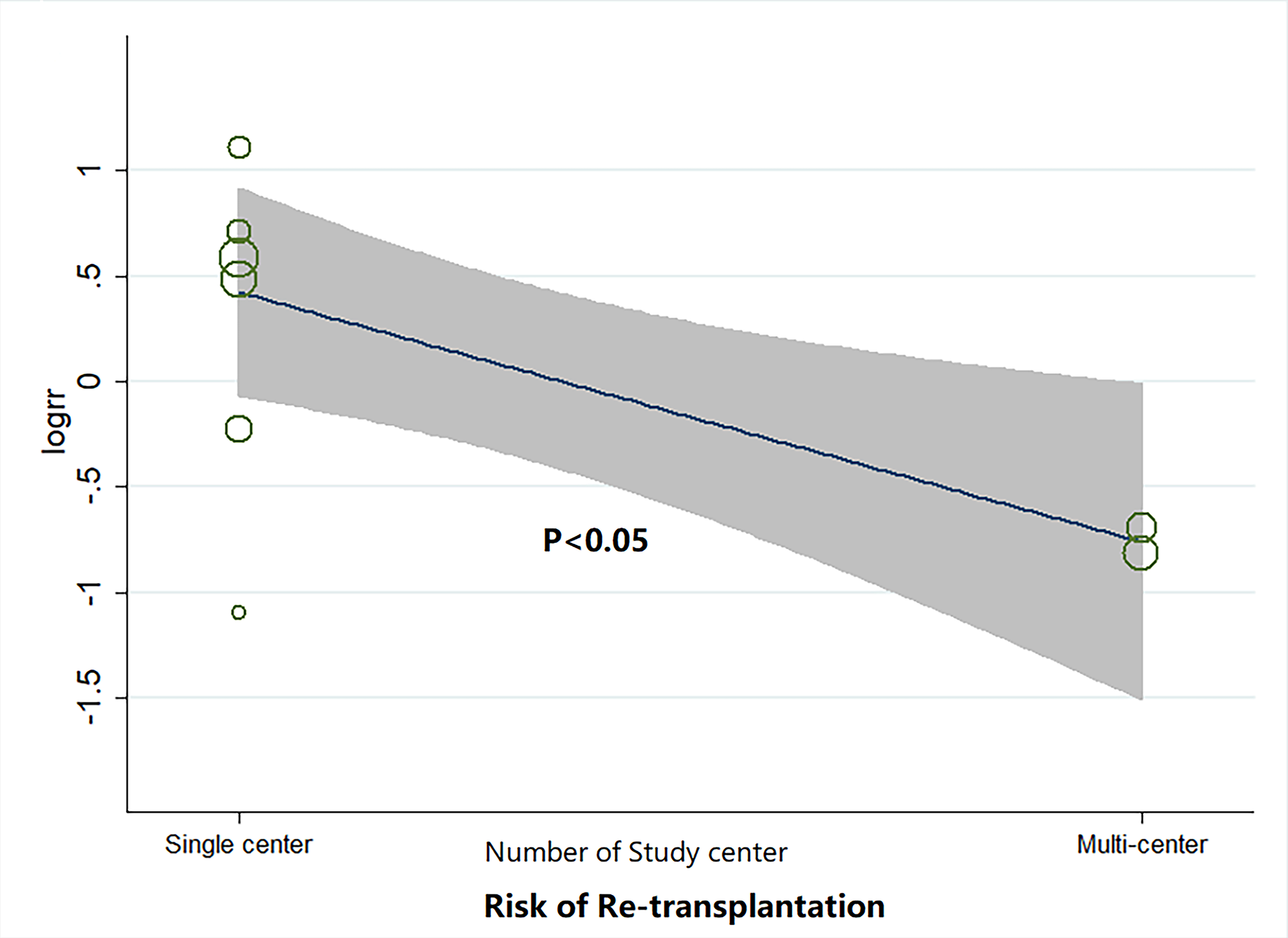

Supplement: Supplementary Figure 8 — Meta regression revealed the impact of center number on donor age related risk of re-transplantation. [file Image_8.TIF]

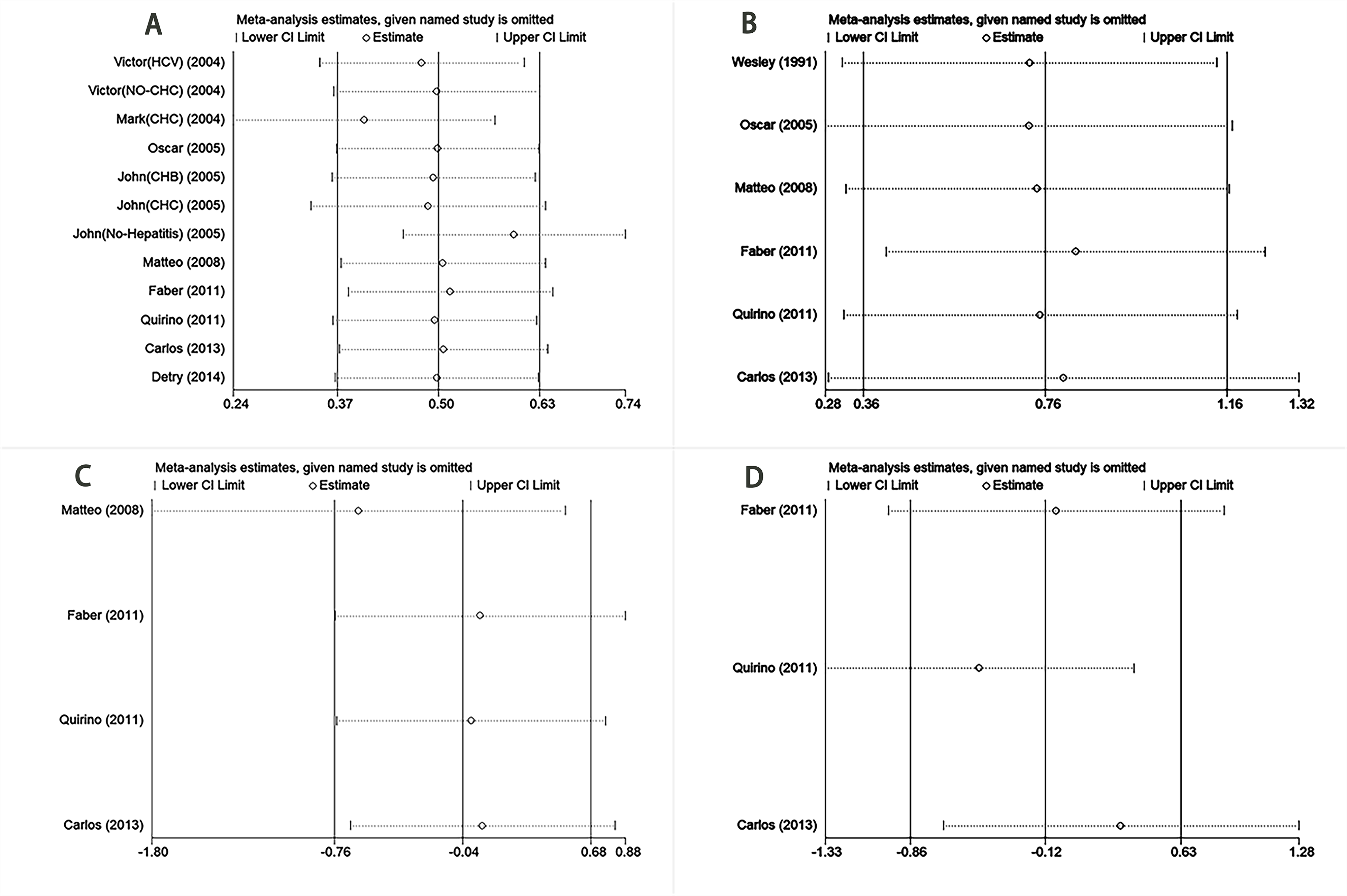

Supplement: Supplementary Figure 9 — Sensitivity analyses on pooled categorical risk of post-transplant outcomes by stepwise omitting each study at a time. Risk was evaluated based on comparison between elder and younger donors. (A) Re-evaluation of donor age related GF risk after omitting each study. (B) Re-evaluation of donor age related risk of patient mortality after omitting each study. (C) Re-evaluation of donor age related risk of PNF after omitting each study. (D) Re-evaluation of donor age related risk of re-transplantation after omitting each study. GF, graft failure; PNF, primary non-function. [file Image_9.TIF]

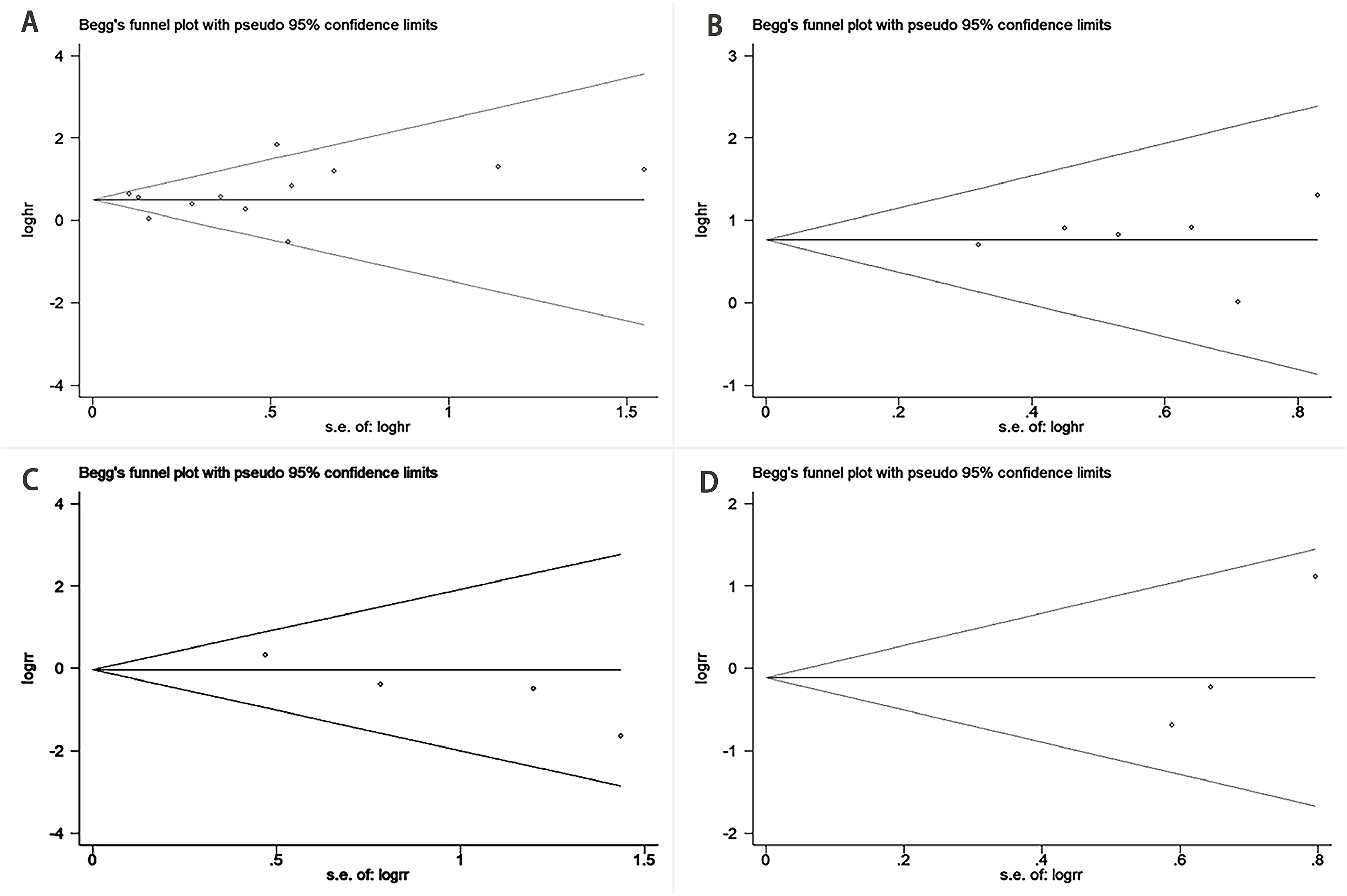

Supplement: Supplementary Figure 10 — Funnel plot analysis on publication bias of categorical risks of aging on post-transplant outcomes. Risk of post-transplant outcomes was compared in recipients using grafts from elder and younger donors. (A) Begg's funnel plot on publication bias of risks on donor age related GF; P for egger's test = 0.66. (B) Begg's funnel plot on publication bias of risks on donor age related patient mortality; P for egger's test = 0.89. (C) Begg's funnel plot on publication bias of risks on donor age related PNF; P for egger's test = 0.05. (D) Begg's funnel plot on publication bias of risks on donor age related re-transplantation; P for egger's test = 0.01. HR, hazard ratio; GF, graft failure; RR, relative risk; PNF, primary non-function. [file Image_10.TIF]

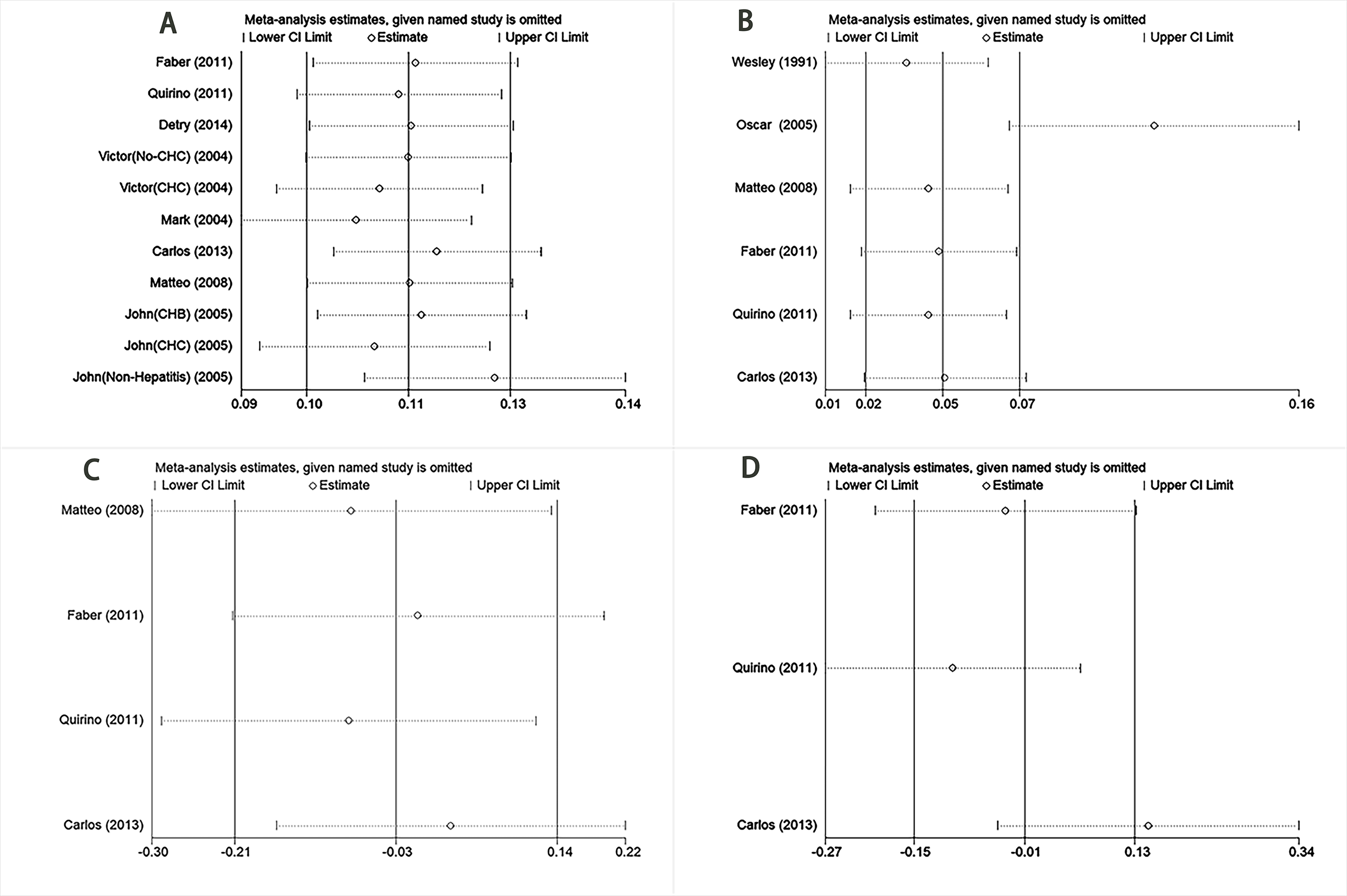

Supplement: Supplementary Figure 11 — Sensitivity analysis on continuous aging related risk by stepwise omitting one study at a time. Risk was evaluated followed per 10-year of donor age increment. (A) Sensitivity analysis on continuous aging related GF risk by stepwise omitting one study at a time. (B) Sensitivity analysis on continuous aging related risk of patient death by stepwise omitting one study at a time. (C) Sensitivity analysis on continuous aging related PNF risk by stepwise omitting one study at a time. (D) Sensitivity analysis on continuous aging related risk of re-transplantation by stepwise omitting one study at a time. GF, graft failure. [file Image_11.TIF]

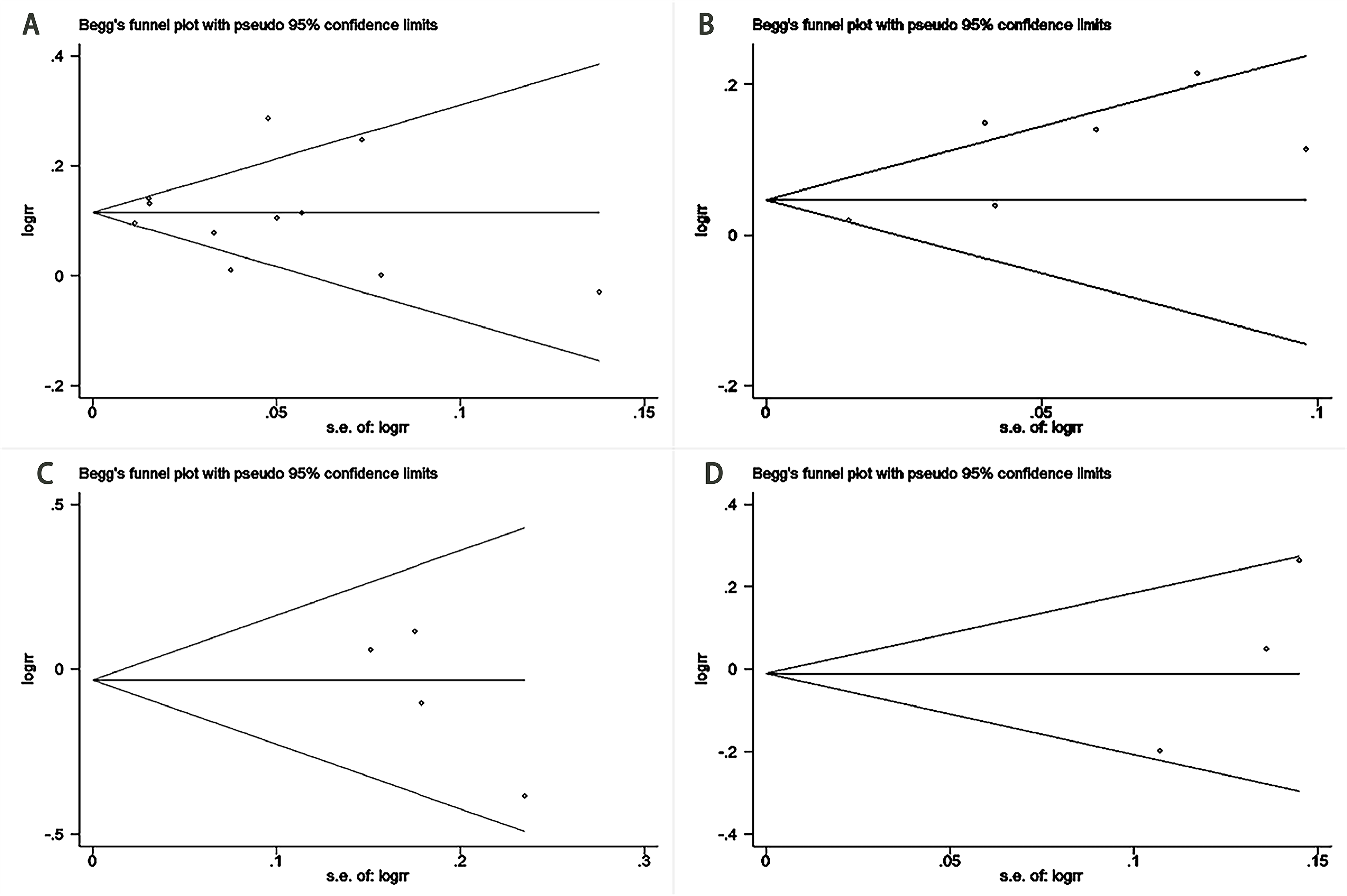

Supplement: Supplementary Figure 12 — Funnel plot analysis on publication bias of continuous risks of aging on post-transplant outcomes. Risk was evaluated followed per 10-year of donor age increment. (A) Begg's funnel plot on publication bias of continuous risks of GF; P for egger's test = 0.98. (B) Begg's funnel plot on publication bias of continuous risks of patient death; P for egger's test = 0.05. (C) Begg's funnel plot on publication bias of continuous risks of PNF; P for egger's test = 0.13. (D) Begg's funnel plot on publication bias of continuous risks of re-transplantation; P for egger's test = 0.14. GF, graft failure; RR, relative risk; PNF, primary non-function. [file Image_12.TIF]
